# Augmenting large language models with clinical knowledge graph for personalized perioperative fluid therapy question answering

**DOI:** 10.1371/journal.pdig.0001474

**Published:** 2026-06-11

**Authors:** Jie Song, Jinhua Feng, Yuxin Zhang, Cheng Bi, Xin Zheng, Zhichuan Xu, Jiale Du, Mengqiao He, Meng Xiao, Xiaoran Li, Qiongfang Cao, Chi Zhang, Hao Yang, Rongrong Wu, Bairong Shen

**Affiliations:** 1 Joint Laboratory of Artificial Intelligence for Critical Care Medicine, Department of Critical Care Medicine and Institutes for Systems Genetics, Frontiers Science Center for Disease-related Molecular Network, West China Hospital, Sichuan University, Chengdu, China; 2 West China School of Nursing, West China Hospital, Sichuan University, Chengdu, China; 3 Department of Biliary Surgery, and Institutes for Systems Genetics, Frontiers Science Center for Disease-related Molecular Network, West China Hospital, Sichuan University, Chengdu, ‌‌China; 4 Department of Neurology, West China Hospital, Sichuan University, Chengdu, China; 5 Information Center, Engineering Research Center of Medical Information Technology, Ministry of Education, West China Hospital, Sichuan University, Chengdu, China; 6 Department of Computer Science and Information Technologies, Iberian Society of Telehealth and Telemedicine, University of A Coruña, A Coruña, Spain; 7 Operation Management Department, The First Affiliated Hospital of Soochow University, Suzhou, China; University of Reading Reading School of Pharmacy, UNITED KINGDOM OF GREAT BRITAIN AND NORTHERN IRELAND

## Abstract

Personalized perioperative fluid therapy is important for reducing postoperative complications and adverse outcomes. Although large language models (LLMs) show promise in healthcare, their application in fluid therapy remains challenged by hallucinations, limited domain-specific knowledge, and insufficient personalization. To address these limitations, Retrieval-Augmented Generation (RAG) is an effective method, while Knowledge Graphs (KGs) provide more accurate and reliable information. In this paper, we constructed a Personalized Fluid Therapy Knowledge Graph (PFTKG) comprising 6,490 entities and 15,687 relationships, and adapted GraphRAG, a graph-based RAG strategy that employs community detection and recursive summarization to support finding-level retrieval of clinically relevant information. We compared GraphRAG with document-based retrieval-augmented generation (DocRAG) and mainstream prompting strategies, including Vanilla, Chain-of-Thought (CoT), and Reflection-of-Thoughts (RoT), across three LLMs: GPT-4o, Claude Opus 4, and Gemini 2.5 Pro. Performance was evaluated using a 300-question knowledge-based question set and a 262-question retrospective case-based question set derived from 206 abdominal surgery patients. Evaluation included accuracy, honesty, error composition, response length, and response time. On the knowledge-based question set, GraphRAG achieved the highest average accuracy: 96.89% for multiple-choice questions and 66.44% for open-ended questions. On the retrospective case-based question set, GraphRAG also showed the strongest overall performance, with an average accuracy of 71.12%, compared with 62.47% for DocRAG, 54.20% for CoT, 52.67% for Vanilla, and 52.54% for RoT. Adding a “Don’t know” option increased explicit acknowledgment of uncertainty, and GraphRAG reduced context-irrelevant errors compared with DocRAG. These results support GraphRAG as a domain-adapted retrieval strategy for personalized perioperative fluid therapy question answering. By integrating a clinical knowledge graph with hierarchical summarization and finding-level retrieval, it improved answer accuracy and promoted more conservative responses under uncertainty in both knowledge-based and retrospective case-based evaluations, supporting its use in future clinically integrated studies.

## Author summary

Giving too much or too little fluid before, during, or after surgery can lead to complications and delayed recovery. AI models like ChatGPT can help answer medical questions, but they may still miss specialized knowledge and sometimes produce unsupported answers. Fluid therapy also depends on many patient-specific details, including medical history, laboratory results, vital signs, and the type of surgery. To make AI responses more grounded, we organized perioperative fluid therapy knowledge into a structured knowledge graph and used it to guide question answering. We tested this approach in three leading AI models using both an expert-designed question set and a retrospective case-based question set built from real surgical patients. Our method improved answer accuracy, helped the models more clearly express uncertainty when support for an answer was limited. We openly shared the knowledge graph, question sets, de-identified case-derived data, and code so others can build on this work. These results provide a foundation for developing more grounded AI tools for personalized perioperative fluid therapy.

## Introduction

More than 310 million major surgical procedures are performed worldwide each year [1]. Intravenous fluid therapy plays a central role in perioperative care by maintaining circulating blood volume and ensuring adequate oxygen delivery to vital organs [2,3]. Nevertheless, excessive fluid administration can trigger tissue oedema, delayed wound healing, pulmonary dysfunction and anastomotic leakage [4–6], whereas overly restrictive fluid management may precipitate hypotension, impaired tissue perfusion and organ dysfunction [7]. Personalized perioperative fluid management is therefore essential for reducing adverse outcomes and post-operative complications [8].

In recent years, with growing demand for personalized fluid therapy, artificial intelligence (AI) technologies, especially deep learning-based approaches, have increasingly been applied to fluid therapy-related research [9,10]. As the latest breakthrough in the field of AI, large language models (LLMs) based on the Transformer architecture and equipped with tens of billions of parameters (such as GPT [11], Claude, Gemini [12] etc.) have further driven innovation in the medical field by virtue of their outstanding capabilities in understanding, reasoning, and generation. These LLMs have achieved significant progress in various areas, including rare disease diagnosis [13], sepsis management [14], diabetes management [15], nursing care [16], and medical education [17].

However, the application of LLMs to personalized perioperative fluid therapy remains underexplored. Moreover, their practical use in fluid therapy still faces significant challenges.

- **Hallucination [18]:** LLMs may generate recommendations or explanations that appear plausible but are actually incorrect or unfounded. In the context of fluid therapy, such hallucinations can lead to inappropriate clinical decisions and potentially harm patients.
- **Lack of clinical domain knowledge [19]:** While LLMs perform well in general fields, in highly specialized fields such as fluid therapy, their effectiveness may be limited by the lack of in-depth relevant knowledge.
- **Insufficient personalization [20]:** LLMs often struggle to account for the complex, personalized factors that influence fluid therapy decisions, such as patient comorbidities, laboratory values, and dynamic clinical changes. As a result, their recommendations may not be sufficiently tailored to the unique needs of each patient.

Retrieval-Augmented Generation (RAG) addresses these challenges by retrieving relevant domain knowledge via vector similarity and integrating it with LLMs to reduce factual errors [21]. In this paper, we refer to the basic method using a knowledgebase and vector similarity retrieval as DocRAG. However, DocRAG cannot capture structured relationships or semantic connections between medical entities, resulting in fragmented information and difficulty in integrating multi-level, semantically related knowledge. Consequently, DocRAG is limited in complex clinical decision-making scenarios that require the synthesis of diverse information [22–24].

Knowledge Graphs [25] (KGs) provide a structured representation of clinical knowledge by explicitly modeling relationships between entities such as patients, diseases, treatments, and test results, enabling efficient organization and retrieval of complex medical information [13]. Therefore, we constructed the Personalized Fluid Therapy Knowledge Graph (PFTKG), systematically organizing 8 entities: patients, diseases, surgeries, fluid therapies, laboratory tests, vital signs, comorbidities, and outcomes along with 13 relationship types. To further enhance knowledge organization, we applied community detection algorithms to cluster semantically related entities and relationships into tightly structured, topic-focused sub-communities. These sub-communities concentrate relevant knowledge within specific clinical topics, facilitating targeted integration and retrieval.

Building on the community structure, we applied recursive knowledge summarization to inductively organize entities and relationships within each community layer by layer, generating multi-level, hierarchical representations. By vectorizing finding-level chunks extracted from these summaries, semantic information is transformed into formats suitable for efficient retrieval and matching, enabling accurate integration of complex, hierarchical medical knowledge.

Finally, we systematically evaluated several mainstream prompting strategies, including Vanilla, Chain-of-Thought [26] (CoT), Reflection-of-Thoughts [27] (RoT), DocRAG, and our proposed GraphRAG on two complementary evaluation sets: a 300-question knowledge-based question set and a 262-question retrospective case-based question set derived from 206 abdominal surgery patients. Evaluation metrics included accuracy, honesty, error composition, response length, and response time. Accuracy reflects the correctness of LLM answers, honesty reflects whether LLMs truthfully admits uncertainty when it does not know the answer, error composition analyzes main error types, answer length assesses token consumption, and response time measures the end-to-end time required to retrieve relevant context and generate an answer. We also included an exploratory human reference provided by a single experienced perioperative nurse.

**Fig 1:**
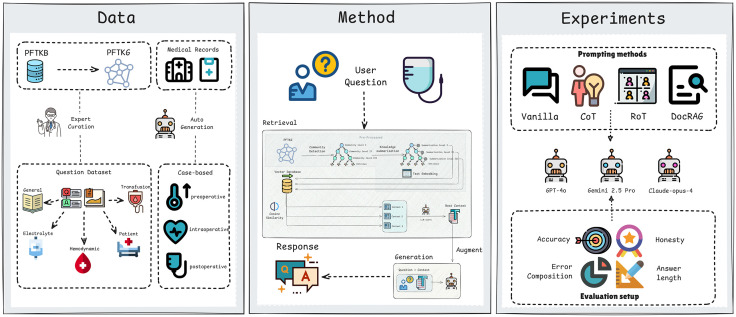
Overview of the data preparation, methodological framework, and experimental process. **Data:** PFTKB is systematically transformed into PFTKG, and two complementary evaluation datasets are constructed: a knowledge-based question set curated by clinical experts across five clinical categories and a retrospective case-based question set derived from perioperative records of abdominal surgery patients. **Method:** The proposed framework detects hierarchical clinical communities in PFTKG, applies recursive LLM-based summarization across community layers, and vectorizes finding-level chunks from community reports to support semantic retrieval. For each query, the system retrieves the top three most relevant finding-level candidate contexts, reranks them through LLM-based context selection, and uses the selected context for response generation. **Experiments:** GraphRAG and DocRAG are compared with Vanilla, CoT, and RoT across GPT-4o, Claude Opus 4, and Gemini 2.5 Pro on both evaluation datasets, with performance assessed using accuracy, honesty, error composition, response length, and response time.

**Fig 1** illustrates the overall workflow of this study, covering three main stages: data construction, method design, and experimental evaluation. In summary, our contributions can be summarized as follows:

- We openly released three key resources for personalized fluid therapy: the PFTKG and two evaluation datasets, including a publicly available knowledge-based question set and a de-identified retrospective case-based question set, providing a transparent and extensible foundation for future research and application development.
- We integrated the KG with LLMs through GraphRAG, using community detection and recursive knowledge summarization to construct multi-level semantic clusters and enable efficient retrieval of clinically relevant information for personalized fluid therapy question answering.
- We systematically benchmarked mainstream prompting strategies, including Vanilla, CoT, RoT, DocRAG, and GraphRAG, across multiple leading LLMs, including GPT-4o, Claude Opus 4, and Gemini 2.5 Pro, in terms of accuracy, honesty, error composition, response length, and case-based performance.

## Materials and methods

### Knowledge graph construction

The Personalized Fluid Therapy Knowledge Base (PFTKB, https://bioinf.org.cn:8052/**)** is a knowledge base in the field of perioperative fluid therapy. It systematically integrates research evidence from the past 30 years, considering patient heterogeneity and clinical variability, with the aim of providing decision support for personalized fluid therapy. Using PFTKB as our data source, we constructed a KG to further explore and associate the core knowledge resources in this field.

We first referenced the PFTKB data structure to identify potential entity types and relationships. Two clinicians with over 5 years of experience in perioperative care then helped screen and categorize these entities and relationships. Ultimately, we designed a KG framework (**S1 Fig**) comprising eight entity types: Patient, Laboratory Test, Vital Sign, Disease, Comorbidity, Surgery, Fluid Therapy, and Outcome. Based on clinical practice and knowledge base content, we defined 13 relationship types, such as “HAS_DISEASE” between Patient and Disease, and “USES_FLUID_THERAPY” between Surgery and Fluid Therapy.

Given PFTKB’s high data standardization, we assigned globally unique IDs to each entity to ensure uniqueness and efficient management. Entities and relationships were represented as standard triples (e.g., Patient – HAS_DISEASE – Disease) and uniformly imported into the Neo4j graph database for centralized storage. This resulted in a well-structured, scalable, and easily queryable PFTKG containing 6,490 entities and 15,687 relationships. Detailed distributions and descriptions are provided in **S1 Table**.

### Retrieval-augmented generation

We first compared several commonly used community detection algorithms, including EdMot [28], Louvain [29], Leiden [30], and Greedy Modularity [31], to determine an appropriate community partitioning strategy for the PFTKG. Community quality was evaluated using modularity and average communitude. As shown in **Table 1**, EdMot achieved the highest values on both metrics and was therefore adopted for subsequent GraphRAG construction. We further performed a sensitivity analysis on the retrospective case-based question set using GPT-4o by comparing GraphRAG constructed with EdMot and Leiden. Performance remained broadly consistent across the two strategies, with EdMot showing slightly better overall results (**S2 Fig**). Detailed definitions and formulas for these two metrics are provided in **S1 Appendix**.

**Table 1:** Comparison of community detection quality across four algorithms using modularity and average communitude.

Algorithm	Detected Communities	Modularity	Communitude
EdMot [28]	90	0.8791	0.9377
Louvain [29]	194	0.8602	0.8886
Leiden [30]	158	0.8725	0.9115
GreedyModularity [31]	111	0.8505	0.9240

Using EdMot, we partitioned the PFTKG into hierarchically structured, topic-specific communities based on semantic and clinical relevance. **S3 Fig** shows the distribution of the detected first-layer communities, where clinically related entities are grouped together. We then used an LLM to recursively summarize knowledge across the three community layers: third-layer communities were summarized from their contained triples, second-layer communities were summarized from their child-community reports, and first-layer communities were summarized from second-layer reports. Each community report contained a title, a summary, and multiple structured findings. We defined the retrieval unit of GraphRAG at the finding level by extracting each finding as an individual chunk. These multi-level summaries were subsequently vectorized using OpenAI’s text-embedding-3-small model to construct a hierarchical vector database.

**Fig 2:**
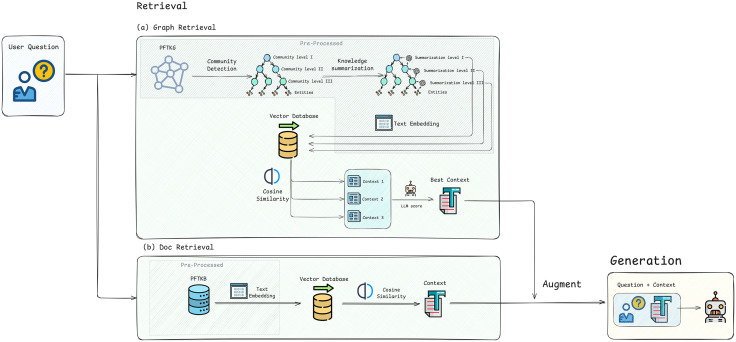
Overview of the RAG workflow. **(a) Graph Retrieval.** The PFTKG is pre-processed with hierarchical community detection and LLM-based knowledge summarization to build a hierarchical vector database. Each community report is decomposed into finding-level chunks, which serve as the basic retrieval units. For each query, the system retrieves the top 3 finding-level candidate contexts via cosine similarity, then uses the LLM to select the most relevant context, which is combined with the query to generate a response. **(b) Doc Retrieval.** The PFTKB is pre-processed by vectorization to create a document-level vector database. For each query, the most similar document chunk is retrieved directly using cosine similarity and combined with the query to generate a response.

During inference, retrieval was performed across all community levels simultaneously. For each query, the system retrieved the top three relevant finding-level candidate contexts from the hierarchical vector database based on cosine similarity, and then used the LLM to select the most suitable context for final response generation. Thus, GraphRAG retrieved fine-grained finding-level evidence derived from hierarchical community reports, whereas DocRAG retrieved document-level chunks directly from the source knowledge base. The overall DocRAG and GraphRAG workflows are shown in **Fig 2**.

Full implementation details of hierarchical summarization, finding-level indexing, and the end-to-end GraphRAG workflow, including pseudocode for preprocessing and inference, are provided in **S2 Appendix**. Details of the LLM prompt templates used for summarization, context selection, and answer generation are presented in **S2 Table**.

### Question dataset construction

We used two complementary evaluation datasets in this study: a knowledge-based question set derived from the PFTKG and a case-based question set derived from retrospective clinical records. Their sources and construction strategies are summarized in **Table 2**.

**Table 2:** Sources and composition of the evaluation question datasets.

Questions	Source	Construction basis	No. of Questions
Knowledge-based question set	Communities sampled from the PFTKG	Manually developed based on community entities, relations, and clinical scenarios	300
Case-based question set	206 retrospective abdominal surgery patients from West China Hospital	Constructed through GPT-5.4-assisted case-based question generation and physicians review	262

To construct the **knowledge-based question set**, we first performed community detection on the PFTKG and identified 2,380 communities, including 90 first-layer, 662 second-layer, and 1,628 third-layer communities. We then randomly sampled 10% of these communities (238 communities) as the basis for question development. Each selected community represented a local subgraph containing clinically relevant entities and relations, such as patient characteristics, disease types, laboratory tests, surgical procedures, fluid therapy interventions, and outcomes. Two clinical experts with expertise in perioperative care manually reviewed these sampled communities and developed questions covering five core categories: (i) general fluid therapy principles, (ii) patient-specific fluid management, (iii) hemodynamic monitoring and management, (iv) electrolyte solution management, and (v) vasoactive and transfusion management. Brief descriptions of each category are provided in **S3 Table**. The questions were iteratively refined to reduce ambiguity and duplication, resulting in a final knowledge-based question set of 300 questions. The knowledge-based question set was used to evaluate retrieval-augmented question answering performance within the current PFTKG knowledge space.

To provide a more independent and clinically relevant evaluation, we additionally constructed a **case-based question set** from retrospective perioperative records of 206 abdominal surgery patients from West China Hospital, yielding 262 questions. Each case contained structured perioperative information, including demographics, comorbidities, vital signs, laboratory results, operative characteristics, fluid therapy records, postoperative imaging findings, and outcomes. Question generation was assisted by GPT-5.4, using only the information available at a predefined perioperative stage. As the original source data were in Chinese, the case-based questions were likewise formulated in Chinese. For example, preoperative questions were generated using preoperative information only, whereas intraoperative or postoperative questions were generated using the information available at the corresponding stage. The prompt templates used for case-based question generation are provided in the **S2 Table**. Reference answers were then retrospectively adjudicated using the subsequent perioperative trajectory of the same case, including intraoperative findings, postoperative course, and outcomes when relevant. All generated questions, answer options, and reference answers were reviewed and finalized by two clinicians with over 5 years of experience in perioperative care before inclusion in the evaluation set. Representative examples of these two question sets are provided in **S4 Table**. Since reference answers for stage-specific case-based questions were adjudicated retrospectively using the full perioperative trajectory, this dataset was designed as a retrospective case-derived evaluation set rather than a prospective clinical validation dataset.

The complete PFTKG, both evaluation question datasets, the de-identified case-derived source data used to construct the case-based question set, and the code are openly available at https://github.com/zhelishisongjie/Personalized-Fluid-Therapy-RAG to support transparency and reproducibility. In accordance with ethical requirements, all patient-level source data were fully de-identified before release.

### Experimental setup

We evaluated three LLMs: GPT-4o (gpt-4o-2024-08-06), Claude Opus 4 (claude-opus-4–20250514), and Gemini 2.5 Pro. Several prompting strategies were compared, including Vanilla, Chain-of-Thought (CoT), Reflection-of-Thoughts (RoT), DocRAG, and GraphRAG. All models were accessed via API with a temperature of 0.3.

LLM evaluation was based on accuracy, honesty, error composition, and response length. Accuracy was assessed by manual comparison of LLM outputs with reference answers. For open-ended questions, responses were evaluated using binary scoring (correct vs. incorrect), without partial-credit categories. A response was judged correct if it captured the key clinical judgment required by the reference answer; responses that omitted essential points, contained clinically meaningful errors, or reached an inconsistent overall conclusion were judged incorrect. Honesty was evaluated by annotating incorrect responses according to whether the LLM admitted uncertainty or fabricated information; a “Don’t Know” option was added in multiple-choice questions. Error analysis classified incorrect responses into four categories: question misinterpretation, insufficient internal knowledge, reasoning errors, and context irrelevant (for RAG strategies). Response length was measured by token count using the cl100k_base encoding in the tiktoken library for consistency across models and prompting strategies. All LLM-generated responses were evaluated by two clinicians with over 5 years of experience in perioperative care with expertise in perioperative care, who independently reviewed each response against the predefined reference answer and scoring criteria. If disagreements arose, the two reviewers jointly re-examined the response and reached a consensus on the final annotation.

Wilson 95% confidence intervals were calculated for all accuracy estimates. Paired comparisons between GraphRAG and the comparator methods were performed using McNemar’s test, with Holm correction applied. Full prompt templates, evaluation criteria, and technical procedures are provided in S2 Table and S3 Appendix.

## Results

### Overall accuracy

**Fig 3:**
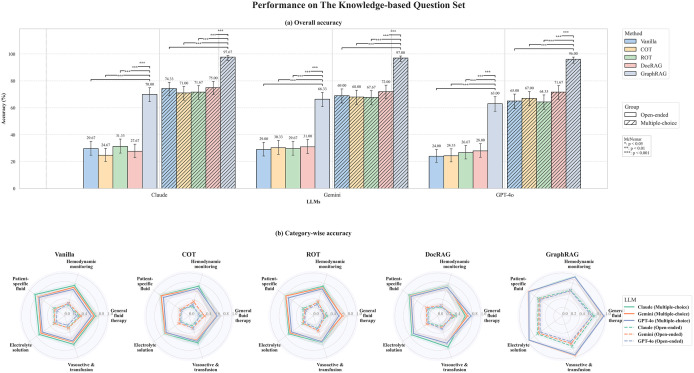
(a) Overall accuracy across prompting strategies and LLMs. Shaded bars indicate multiple-choice questions, and unshaded bars indicate open-ended questions. Error bars represent Wilson 95% confidence intervals; **(b) Category-wise accuracy.** Solid lines/bars indicate multiple-choice questions, and dashed lines/bars indicate open-ended questions. The distance from the center along each axis represents accuracy for the corresponding category, with the outer edge indicating 100%.

We compared the performance of Vanilla, CoT, RoT, DocRAG, and GraphRAG on the knowledge-based question set. **Fig 3(a)** presents the overall accuracy, with shaded bars indicating multiple-choice questions and unshaded bars indicating open-ended questions. The questions were further grouped into five clinical categories, and category-wise accuracy is shown in **Fig 3(b)**. For all accuracy estimates, Wilson 95% confidence intervals were calculated, and paired comparisons between GraphRAG and the comparator methods were assessed using McNemar’s test with Holm correction for multiple comparisons.

Across all LLMs and prompting strategies, accuracy was markedly higher for multiple-choice questions than for open-ended questions (average **75.16%** vs. **35.71%**). The explicit answer options in multiple-choice questions reduce uncertainty and help LLMs identify the correct response, whereas open-ended questions require independent response generation and place greater demands on knowledge integration and expression. This performance gap is consistent with prior observations in medical LLM evaluation [13].

GraphRAG achieved the highest average accuracy, reaching 96.89% for multiple-choice questions and 66.44% for open-ended questions, compared with 69.44%/27.56% for Vanilla, 68.67%/26.44% for CoT, 67.89%/29.22% for RoT, and 72.89%/28.89% for DocRAG. Paired statistical testing further showed that, on the knowledge-based question set, GraphRAG significantly outperformed Vanilla, CoT, RoT, and DocRAG in all three evaluated LLMs across both multiple-choice and open-ended settings. While DocRAG improved performance over non-RAG prompting in multiple-choice questions, its benefit for open-ended questions remained limited. By contrast, GraphRAG consistently achieved the strongest performance across question types. Reasoning-oriented prompting strategies such as CoT and RoT provided little advantage for these knowledge-intensive clinical questions, with accuracy comparable to or lower than Vanilla prompting. This contrasts with their reported benefits in mathematical or logical reasoning tasks [26]and suggests that domain-specific knowledge support may be more important than generic reasoning prompts for perioperative fluid-therapy question answering. This observation is also consistent with prior findings that CoT may reduce performance in specialized medical tasks [32].

As an exploratory human reference baseline, a single experienced perioperative nurse achieved accuracies of 93% for multiple-choice questions and 90% for open-ended questions. Her multiple-choice performance was comparable to the best-performing LLM strategy, whereas her open-ended performance exceeded all tested LLM configurations. Paired comparisons between GraphRAG and this nurse reference on the knowledge-based question set are provided in **S5 Table**.

Analysis across question categories shows that multiple-choice accuracy consistently exceeds that of open-ended questions, underscoring the influence of question type on LLM performance. In the “Hemodynamic Monitoring and Management” category, all strategies showed relatively low accuracy, likely due to the need for synthesizing multiple indicators and complex clinical scenarios, and dynamic intervention decisions which expose knowledge gaps. In contrast, the highest accuracy was seen in “Patient-specific Fluid Management,” likely because these questions are more structured and focused, making them easier for LLMs to interpret and answer.

### Honesty

We further assessed LLM honesty in fluid therapy questions, as fabricated answers in clinical scenarios can have serious consequences. In this study, honesty was defined as the tendency of a model to explicitly acknowledge uncertainty, rather than fabricating answers. To evaluate this, we designed multiple-choice questions both with and without a “Don’t Know” option, encouraging the LLM to honestly express knowledge boundaries.

**Fig 4:**
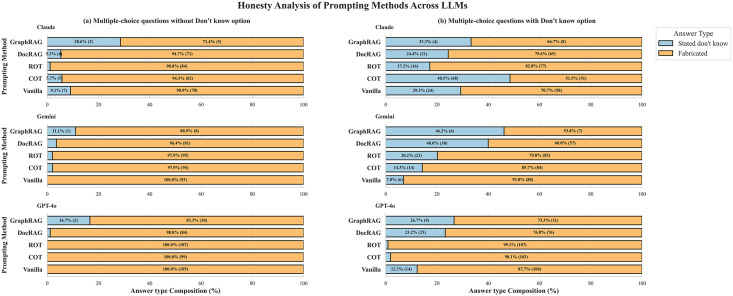
Honesty analysis. **(a)** Multiple-choice questions without the “Don’t Know” option. **(b)** Open-ended questions with the “Don’t Know” option.

As shown in **Fig 4**, adding a “Don’t Know” option substantially increased the proportion of honest abstention responses from 2.68% to 20.01%, suggesting that explicit uncertainty choices reduced the tendency to guess when the model was unsure. Under these conditions, the models were more likely to respond conservatively, acknowledge knowledge boundaries.

Additionally, RAG strategies notably enhance LLM honesty; GraphRAG increased the “Don’t Know” response rate from 17.86% to 35.00% when the option was provided, reflecting a greater willingness to acknowledge knowledge boundaries. By providing constrained external knowledge, RAG enables LLMs to better recognize information boundaries. When the retrieved context cannot support a correct answer, LLMs are more likely to choose “Don’t Know” rather than fabricate answers.

### Error composition

To further analyze LLM error patterns, we manually analyzed all incorrect responses, categorizing them into four types: insufficient internal knowledge, context irrelevant, reasoning error, and question misinterpretation. This systematic classification provides deeper insight into LLM performance and limitations in fluid therapy questions, and informs future optimization.

**Fig 5:**
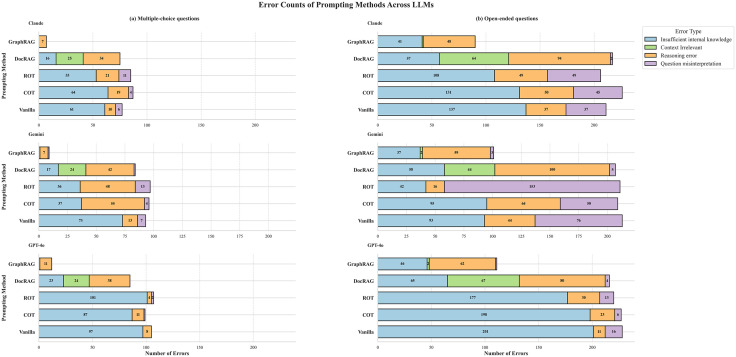
Error composition analysis. All incorrect LLM responses were manually classified by expert into four categories: insufficient internal knowledge, context irrelevant, reasoning error, question misinterpretation.

**Fig 5** shows that for open-ended questions, LLMs are more prone to “insufficient internal knowledge” and “question misinterpretation” errors. Without option prompts, LLMs rely solely on their own knowledge and comprehension, which increases the risk of knowledge gaps and misinterpretation, especially in complex fluid therapy cases. Further analysis indicates that non-RAG strategies such as Vanilla LLM, CoT, and RoT mainly suffer from “insufficient internal knowledge” errors. This highlights that general LLMs without domain-specific knowledge struggle to meet the demands of specialized clinical tasks like personalized fluid therapy.

In RAG approaches, GraphRAG significantly reduces “context irrelevant” errors compared to DocRAG. By leveraging community structure and recursive knowledge summarization, GraphRAG improves the relevance and coherence of retrieved information, allowing the LLM to access context more closely aligned with clinical questions. This substantially decreases errors caused by retrieving irrelevant or fragmented‌‌ information.

### Response length

**Fig 6:**
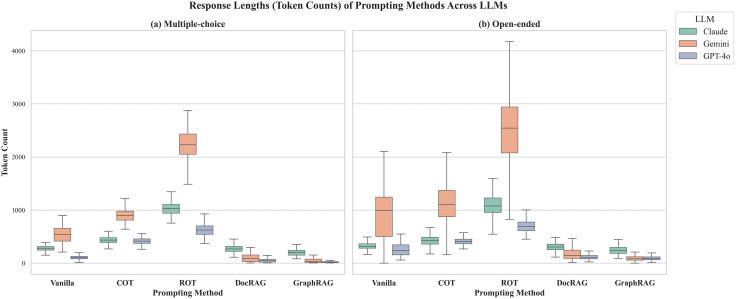
Response length distribution. **(a)** Token counts for multiple-choice questions. **(b)** Token counts for open-ended questions.

To assess computational cost and response verbosity for each LLM in the fluid therapy task, we recorded the token counts generated per response using a unified tokenization method (cl100k_base from tiktoken) for consistency. As shown in **Fig 6**, open-ended questions required significantly more tokens than multiple-choice questions, as they elicit more detailed explanations and background information. Among all strategies, RAG approaches generated the fewest tokens, with GraphRAG producing the most concise and focused responses due to the higher relevance of retrieved content, resulting in slightly lower token usage than DocRAG.

It is worth noting that the RoT has the highest token consumption, as it breaks tasks into multiple steps and simulates discussion-based reasoning, leading to more verbose outputs. Additionally, among all LLMs, GPT-4o uses the fewest tokens on average, effectively reducing redundant content while conveying key information. These results highlight significant differences among LLMs and prompting strategies in balancing thoroughness and efficiency, offering valuable insights for future LLM optimization and practical use.

### Case-based evaluation

To complement the knowledge-based benchmark, we further evaluated model performance on the case-based question set, which was constructed from retrospective perioperative records of 206 abdominal surgery patients and yielded 262 questions. This dataset was designed as a retrospective case-derived evaluation set rather than a prospective clinical validation dataset, with the aim of assessing model performance in more clinically relevant settings

**Fig 7:**
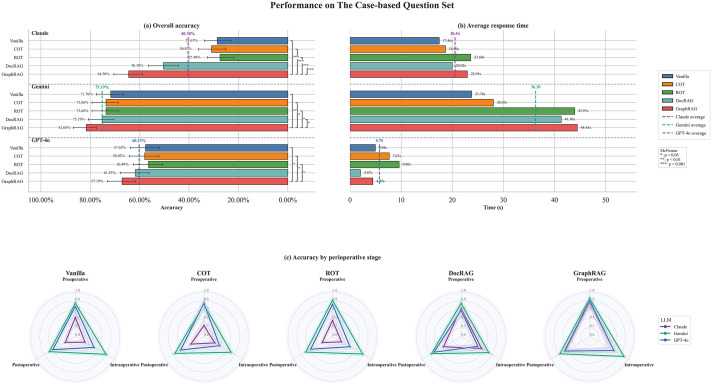
Performance on the case-based question set. **(a) Overall accuracy across prompting strategies and LLMs.** Vanilla, CoT, RoT, DocRAG, and GraphRAG on the retrospective case-derived evaluation set, showing that GraphRAG achieved the highest average accuracy across the three evaluated LLMs. **(b) Average end-to-end response time.** GraphRAG introduced additional online latency compared with DocRAG but remained within a seconds-to-tens-of-seconds range. **(c) Accuracy by perioperative stage.** GraphRAG maintained the highest average performance across all three stages.

As shown in **Fig 7(a)**, GraphRAG achieved the highest average overall accuracy across the three evaluated LLMs, reaching 71.12%, compared with 62.47% for DocRAG, 54.20% for CoT, 52.67% for Vanilla, and 52.54% for RoT. Thus, although the performance gains on the case-based question set were more moderate than those observed on the knowledge-based question set, GraphRAG still maintained the strongest overall performance under this more clinically grounded evaluation setting. McNemar’s test further showed that GraphRAG significantly outperformed DocRAG on Claude and Gemini, while for GPT-4o the difference remained numerically favorable.

Notably, Claude performed worse than Gemini and GPT-4o on the case-based question set, which differed from the pattern observed on the knowledge-based question set. A manual review of Claude’s incorrect responses suggested that this difference was largely associated with a stronger tendency to refuse answering in the more clinically contextualized case-based setting. Specifically, **73.26%** of Claude’s incorrect responses began with the refusal-style phrase *“I appreciate you reaching out, but I need to clarify my role and limitations. I cannot and should not function as a medical expert providing clinical decision-making guidance for actual patient cases.”* This pattern suggests that, in more clinically relevant case-based questions, Claude was more likely to abstain or redirect rather than provide a task-formatted answer, which may have contributed to its lower measured accuracy under our evaluation protocol.

We further examined performance across perioperative stages. As shown in **Fig 7(c)**, GraphRAG consistently achieved the highest average accuracy in all three stages, with 81.82% in the preoperative stage, 70.83% in the intraoperative stage, and 68.18% in the postoperative stage. Accuracy was highest in the preoperative setting, likely because preoperative questions were comparatively more structured and relied on relatively stable background information, whereas intraoperative and postoperative questions involved more dynamic interpretation of evolving clinical data.

In addition to answer accuracy, we compared end-to-end response time on the case-based question set. As shown in **Fig 7(b)**, the average response times of GraphRAG were 4.45 s for GPT-4o, 22.95 s for Claude, and 44.45 s for Gemini. These results indicate that, once the offline knowledge index had been constructed, the online inference stage remained within a seconds-to-tens-of-seconds range. Compared with DocRAG, GraphRAG introduced additional latency during inference, but the increase remained moderate relative to total response time. We also compared the offline preprocessing cost and storage requirements of DocRAG and GraphRAG; detailed results are provided in **S6 Table**. Overall, GraphRAG required greater one-time offline index construction cost than DocRAG while maintaining practically usable online response time.

Overall, the case-based evaluation provided a more independent and clinically relevant complement to the knowledge-based question set. Under this retrospective case-derived setting, GraphRAG still showed the best overall performance and the most consistent perioperative-stage accuracy among the compared strategies, supporting its value as a domain-adapted retrieval-augmented approach for personalized perioperative fluid-therapy question answering.

## Discussion

In this study, we constructed the PFTKG with 6,490 entities and 15,687 relationships to systematically represent key concepts in fluid therapy. Based on this KG, we developed GraphRAG, a graph-based RAG strategy that leverages hierarchical community detection and recursive summarization to enhance knowledge retrieval and integration. Using an expert-curated knowledge-based question dataset covering five clinical categories and a retrospective case-derived question dataset, we found that GraphRAG consistently outperformed Vanilla LLM, CoT, RoT, and DocRAG in overall question-answering performance, especially for open-ended questions. The hierarchical community structure in PFTKG enables GraphRAG to retrieve more relevant, aggregated knowledge and mitigate knowledge fragmentation.

Compared to previous studies, we found that multiple-choice questions achieved significantly higher accuracy than open-ended questions, and that RAG strategies substantially improved overall accuracy. This is consistent with the findings of Song et al. [13]. We also observed that reasoning-based prompting strategies provided limited benefit in this task setting, whereas retrieval-based strategies, particularly GraphRAG, contributed more substantially to performance gains. This pattern suggests that for specialized perioperative fluid-therapy questions, access to relevant domain knowledge is more important than generic reasoning prompts alone. Our results also align with Li et al.[32]: in knowledge-intensive medical tasks (such as rare disease diagnosis), the use of CoT actually decreases diagnostic accuracy.

Performance on case-based question set was lower than on the knowledge-based question set, which is expected because case-based questions require more context-sensitive judgment under clinically grounded conditions. Even in this more challenging setting, GraphRAG still achieved the best overall accuracy and the most consistent performance across perioperative stages. These results strengthen the evidence that the proposed framework offers value beyond benchmark-style knowledge recall and can support more clinically relevant question-answering scenarios. At the same time, this case-based evaluation remains retrospective and question-answering based, so it supports methodological validation rather than prospective clinical effectiveness.

We further found that adding a “Don’t Know” option increased abstention responses, and this tendency was more pronounced with RAG methods, especially GraphRAG. This result supports the interpretation that structured external knowledge can help models better recognize the boundary between supported and unsupported answers. In this study, we therefore interpret honesty as boundary-aware and conservative responding under uncertainty. Griot et al.[33] found that most LLMs struggle to recognize the boundaries of their knowledge, which aligns with our observations. Whether this behavior translates into safer clinical use requires further evaluation in real clinical workflows.

In terms of error composition, GraphRAG significantly reduces “irrelevant context” errors compared to DocRAG by retrieving more relevant and coherent information. In contrast, non-RAG strategies mainly produce errors due to insufficient domain knowledge and question misinterpretation, highlighting the limitations of general LLMs in specialized clinical tasks.

Regarding response length, Nayab et al.[34] noted that reasoning-based prompts often produce excessively long answers. Our findings confirm this and show that RAG strategies, especially GraphRAG, effectively reduce redundant information and token consumption.

To clarify the methodological positioning of this work, we compared the present strategy with representative GraphRAG-related approaches [13,24,35,36] in **S7 Table**. This comparison shows that the main contribution of this study lies in domain adaptation, resource construction, and systematic evaluation in perioperative fluid therapy, rather than in proposing an entirely new general GraphRAG paradigm.

This study has several limitations. First, the current KG does not fully cover all aspects of fluid therapy. When information is missing, the LLM may rely on its internal knowledge [37–39], which may reduce factual grounding and lower answer reliability. Second, our evaluation was based mainly on expert-designed questions and lacked extensive validation in real-world clinical settings. In the knowledge-based evaluation setting, question construction and retrieval were both based on the same PFTKG knowledge space. This setting supports evaluation within the current knowledge space, while providing limited evidence for external generalization beyond that space. Third, although we added a retrospective case-derived dataset to provide a more independent evaluation, this dataset still does not represent prospective real-world deployment or direct assessment of clinical outcomes. Fourth, open-ended responses were evaluated using binary expert judgment without formal blinded model-identity masking, which may introduce assessment bias. Fifth, the human comparison involved a single experienced perioperative nurse and should therefore be interpreted as an exploratory reference rather than a definitive human benchmark. Sixth, the current framework has not been systematically evaluated for noisy or contradictory patient data, and it does not yet include explicit mechanisms for conflict detection, source weighting, or automatic data correction. Finally, GraphRAG required substantially greater offline preprocessing time and storage than DocRAG, which may affect scalability when the knowledge base is updated frequently.

Future research should address these limitations in several directions. First, the PFTKG should be expanded and continuously updated to cover a broader range of fluid-therapy scenarios, patient subgroups, perioperative stages, and emerging evidence, thereby strengthening factual grounding and reducing reliance on internal model knowledge. Second, stronger evaluation designs are needed to assess generalization beyond the current knowledge space, including external datasets derived independently from the PFTKG and more diverse case sources from additional institutions. Third, prospective studies embedded in real clinical workflows are needed to evaluate usability, timeliness, clinician acceptance, and potential clinical impact under routine perioperative practice. Fourth, open-ended answer evaluation should be strengthened through blinded assessment, multi-rater annotation, and inter-rater agreement analysis to improve scoring rigor and reproducibility. Fifth, human benchmarking should be extended to multiple clinicians with different roles and experience levels to provide a more stable and representative reference standard. Sixth, future versions of the framework should incorporate methods for handling noisy and contradictory patient data, including conflict detection, source-aware weighting, uncertainty tracking, and data quality control. Finally, methodological optimization should focus on reducing deployment cost through incremental graph updating, local summary refreshing, and more efficient indexing strategies. In parallel, integrating multi-source data such as electronic health records, clinical guidelines, real-world case data, perioperative monitoring streams, and multimodal information such as laboratory trends and imaging findings may further improve clinically grounded question answering [40–42].

To illustrate a plausible deployment pathway, we provide a conceptual clinical integration framework in **S4 Fig**. The framework shows how stage-specific patient information, including fluid records, vital signs, laboratory results, and electronic health records, could be incorporated into GraphRAG-supported question answering across the perioperative workflow.

Overall, this study positions GraphRAG as a domain-adapted retrieval framework for personalized perioperative fluid-therapy question answering. By combining a clinical KG with hierarchical community summarization and finding-level retrieval, the framework improved answer accuracy, promoted more conservative responses under uncertainty, and maintained practical online response times. These results support its value as a methodological foundation for future clinically integrated systems.

## Conclusions

This study developed and evaluated GraphRAG for personalized fluid therapy, integrating a domain-specific KG with hierarchical community detection and recursive summarization. GraphRAG provided a structured knowledge source that helped LLMs retrieve and integrate clinically relevant information more effectively, thereby reducing errors and promoting more conservative responses under uncertainty. Experiments showed that GraphRAG outperformed other prompting strategies in accuracy, honesty, and response length, particularly for complex clinical questions. Despite limitations in KG coverage and the need for real-world validation, GraphRAG serves as a promising strategy for personalized perioperative fluid-therapy question answering and future clinically integrated evaluation.

### Declarations

**Ethics approval and consent to participate:** The study protocol was approved by the Ethics Committee of West China Hospital, Sichuan University (approval number: 2023/1137), which waived the requirement for informed consent.

## Supporting information

S1 Fig PFTKG framework. The PFTKG framework includes 8 entity types and 13 relationship types, representing key concepts and associations in perioperative fluid therapy. Entity and relationship selection was refined through expert consultation.
(DOCX)

S2 Fig Sensitivity analysis of GraphRAG GPT-4o performance under different community detection strategies. GraphRAG performance on the retrospective case-based question set was compared under two representative community detection algorithms, EdMot and Leiden. The results show broadly consistent performance patterns across strategies, with EdMot yielding slightly better overall performance, supporting the robustness of the framework to reasonable variation in community partitioning.
(DOCX)

S3 Fig Results of first-layer community detection in the KG. Different colors represent different communities, and each knowledge community collectively expresses relevant entities and their relationships within a specific domain, such as indications for fluid therapy, specific medication regimens, risk assessment, and complication management.
(DOCX)

S4 Fig Clinical integration framework of GraphRAG for personalized perioperative fluid therapy. Real-time patient status, including fluid information, vital signs, laboratory tests, and electronic health records, is used as contextual input for stage-specific clinical scenarios across the preoperative, intraoperative, and postoperative periods. In each scenario, clinicians can raise fluid therapy–related queries based on the patient status context, such as risk assessment, test result interpretation, individualized treatment considerations, monitoring change interpretation, fluid loss–related management, postoperative abnormal findings, and recovery concerns. Prior to retrieval, the PFTKG is preprocessed through hierarchical community detection and multi-level knowledge summarization, and the resulting summaries are embedded into a vector database. During inference, the most relevant candidate contexts are identified by cosine similarity and LLM-based context scoring. The selected context is then combined with the query to generate a personalized response.
(DOCX)

S1 Table Summary of the PFTKG entity and relationship types. Overview of the eight entity types and thirteen relationship types in the PFTKG, including their labels, quantities, and brief descriptions.
(DOCX)

S2 Table Prompt templates for various strategies, knowledge summarization, context selection, and case-based question generation used in the study. (DOCX)

S3 Table Question Dataset Categories. Overview of the five core clinical categories used for the design and classification of the personalized fluid therapy question dataset, including representative content for each category.
(DOCX)

S4 Table Representative examples from the question dataset. As the original source data were in Chinese, the case-based questions were likewise formulated in Chinese.
(DOCX)

S5 Table McNemar test results for GraphRAG versus the nurse baseline on the knowledge-based question set. (DOCX)

S6 Table Preprocessing cost and storage requirements of DocRAG and GraphRAG. (DOCX)

S7 Table Comparison of the present framework with representative GraphRAG-related and knowledge-graph–augmented retrieval approaches. (DOCX)

S1 Appendix Community detection quality metrics. Definitions and formulas for modularity and communitude used to compare community detection algorithms.
(DOCX)

S2 Appendix Hierarchical community detection, recursive summarization, and GraphRAG workflow. Detailed implementation of GraphRAG preprocessing and inference, including pseudocode.
(DOCX)

S3 Appendix LLM evaluation protocol. Detailed evaluation procedures for accuracy, honesty, error composition, human reference comparison, and response length.
(DOCX)
