# Supplementary material for: Augmenting large language models with clinical knowledge graph for personalized perioperative fluid therapy question answering
Source: PLOS Digit Health. 2026 Jun 11;5(6):e0001474. doi: 10.1371/journal.pdig.0001474 (PMC13257993; doi:10.1371/journal.pdig.0001474)
Supplement: S1 Fig — The PFTKG framework includes 8 entity types and 13 relationship types, representing key concepts and associations in perioperative fluid therapy. Entity and relationship selection was refined through expert consultation. (DOCX) [file pdig.0001474.s001.docx]

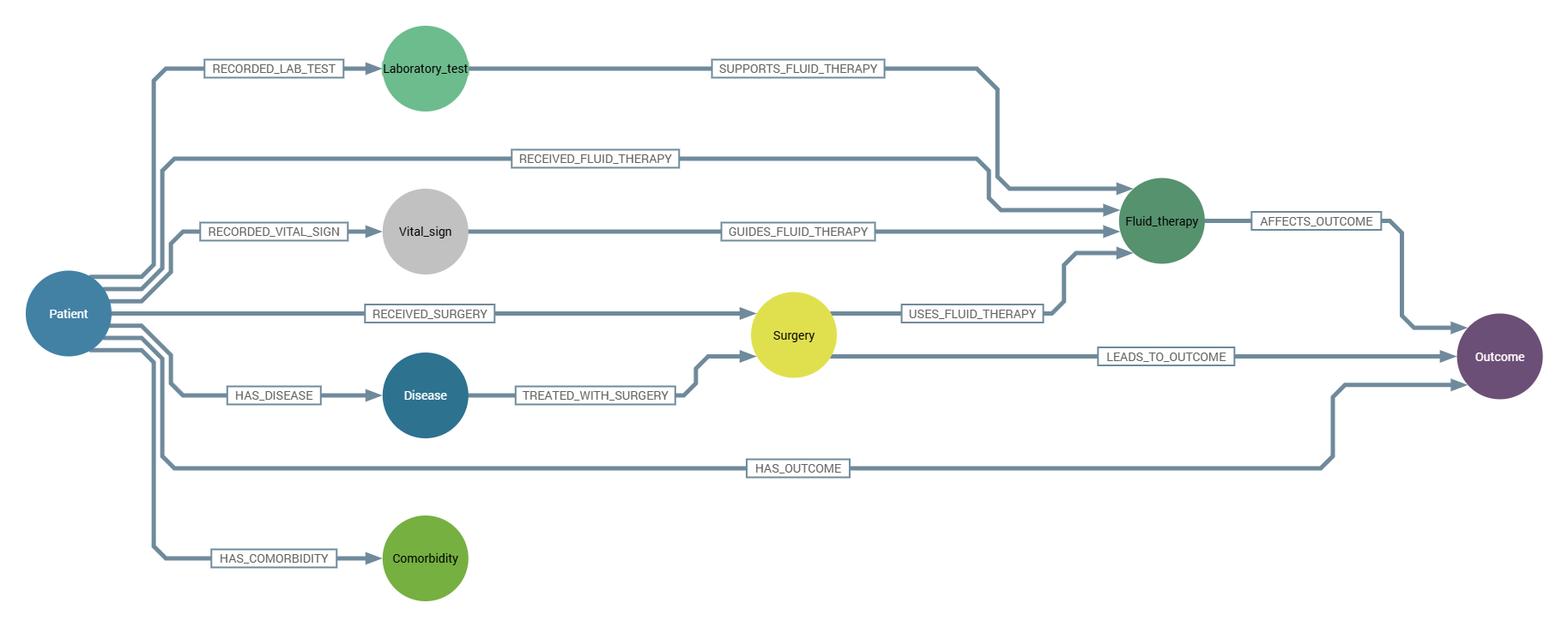


**S1 Fig.** **PFTKG framework.** The PFTKG framework includes 8 entity types and 13 relationship types, representing key concepts and associations in perioperative fluid therapy. Entity and relationship selection was refined through expert consultation.
