# Supplementary material for: Augmenting large language models with clinical knowledge graph for personalized perioperative fluid therapy question answering
Source: PLOS Digit Health. 2026 Jun 11;5(6):e0001474. doi: 10.1371/journal.pdig.0001474 (PMC13257993; doi:10.1371/journal.pdig.0001474)
Supplement: S2 Fig — GraphRAG performance on the retrospective case-based question set was compared under two representative community detection algorithms, EdMot and Leiden. The results show broadly consistent performance patterns across strategies, with EdMot yielding slightly better overall performance, supporting the robustness of the framework to reasonable variation in community partitioning. (DOCX) [file pdig.0001474.s002.docx]

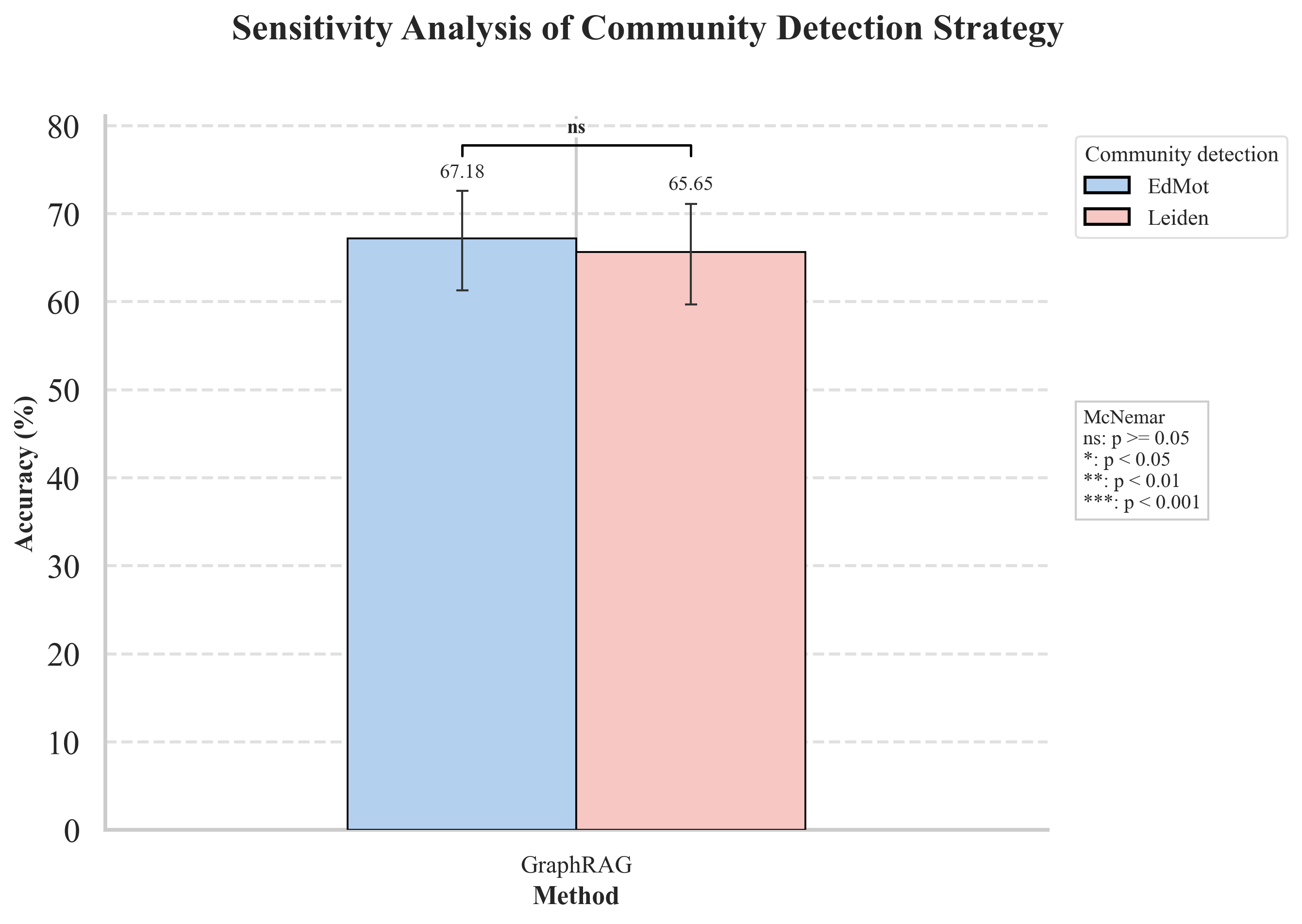


**S2 Fig. Sensitivity analysis of GraphRAG GPT-4o** **performance under different community detection strategies.** GraphRAG performance on the retrospective case-based question set was compared under two representative community detection algorithms, EdMot and Leiden. The results show broadly consistent performance patterns across strategies, with EdMot yielding slightly better overall performance, supporting the robustness of the framework to reasonable variation in community partitioning.
