# Supplementary material for: Augmenting large language models with clinical knowledge graph for personalized perioperative fluid therapy question answering
Source: PLOS Digit Health. 2026 Jun 11;5(6):e0001474. doi: 10.1371/journal.pdig.0001474 (PMC13257993; doi:10.1371/journal.pdig.0001474)
Supplement: S3 Fig — Different colors represent different communities, and each knowledge community collectively expresses relevant entities and their relationships within a specific domain, such as indications for fluid therapy, specific medication regimens, risk assessment, and complication management. (DOCX) [file pdig.0001474.s003.docx]

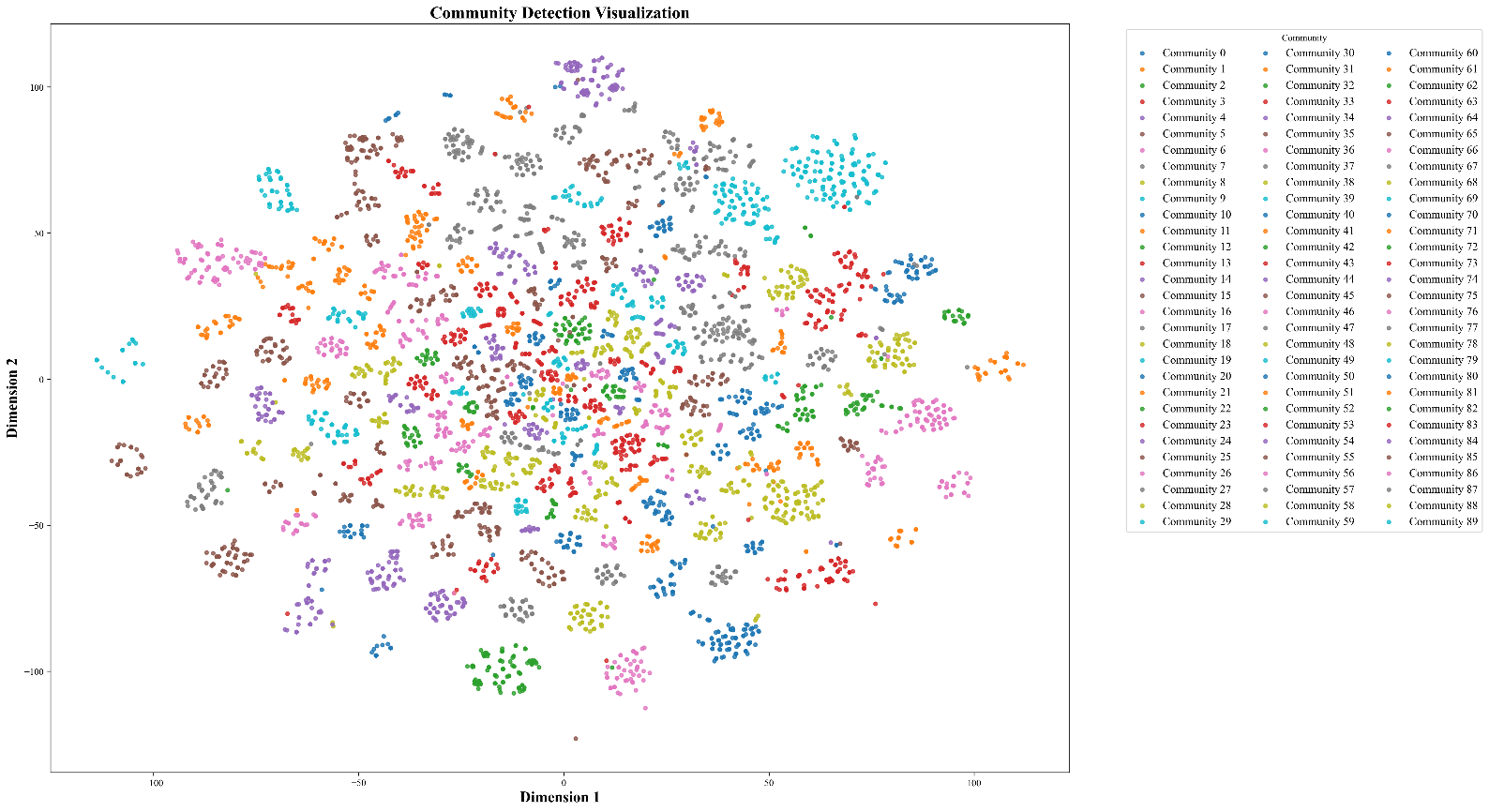


**S3 Fig. Results of first-layer community detection in the KG.** Different colors represent different communities, and each knowledge community collectively expresses relevant entities and their relationships within a specific domain, such as indications for fluid therapy, specific medication regimens, risk assessment, and complication management.
