# Supplementary material for: Augmenting large language models with clinical knowledge graph for personalized perioperative fluid therapy question answering
Source: PLOS Digit Health. 2026 Jun 11;5(6):e0001474. doi: 10.1371/journal.pdig.0001474 (PMC13257993; doi:10.1371/journal.pdig.0001474)
Supplement: S4 Fig — Real-time patient status, including fluid information, vital signs, laboratory tests, and electronic health records, is used as contextual input for stage-specific clinical scenarios across the preoperative, intraoperative, and postoperative periods. In each scenario, clinicians can raise fluid therapy–related queries based on the patient status context, such as risk assessment, test result interpretation, individualized treatment considerations, monitoring change interpretation, fluid loss–related management, postoperative abnormal findings, and recovery concerns. Prior to retrieval, the PFTKG is preprocessed through hierarchical community detection and multi-level knowledge summarization, and the resulting summaries are embedded into a vector database. During inference, the most relevant candidate contexts are identified by cosine similarity and LLM-based context scoring. The selected context is then combined with the query to generate a personalized response. (DOCX) [file pdig.0001474.s004.docx]

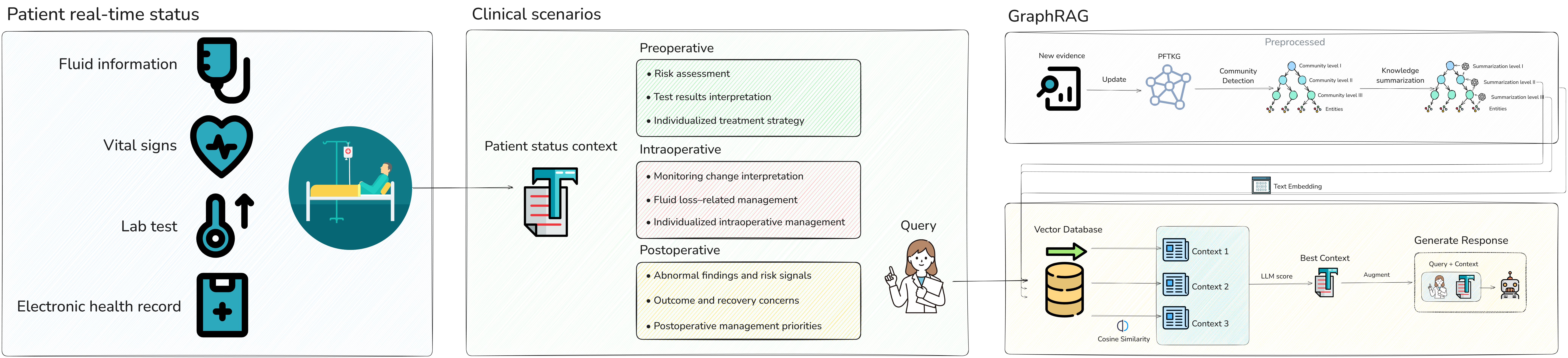


**S4 Fig. Clinical integration framework of GraphRAG for personalized perioperative fluid therapy.** Real-time patient status, including fluid information, vital signs, laboratory tests, and electronic health records, is used as contextual input for stage-specific clinical scenarios across the preoperative, intraoperative, and postoperative periods. In each scenario, clinicians can raise fluid therapy–related queries based on the patient status context, such as risk assessment, test result interpretation, individualized treatment considerations, monitoring change interpretation, fluid loss–related management, postoperative abnormal findings, and recovery concerns. Prior to retrieval, the PFTKG is preprocessed through hierarchical community detection and multi-level knowledge summarization, and the resulting summaries are embedded into a vector database. During inference, the most relevant candidate contexts are identified by cosine similarity and LLM-based context scoring. The selected context is then combined with the query to generate a personalized response.
