# Supplementary material for: Augmenting large language models with clinical knowledge graph for personalized perioperative fluid therapy question answering
Source: PLOS Digit Health. 2026 Jun 11;5(6):e0001474. doi: 10.1371/journal.pdig.0001474 (PMC13257993; doi:10.1371/journal.pdig.0001474)
Supplement: S1 Table — Overview of the eight entity types and thirteen relationship types in the PFTKG, including their labels, quantities, and brief descriptions. (DOCX) [file pdig.0001474.s005.docx]

**S1 Table. Summary of the PFTKG entity and relationship types.** Overview of the eight entity types and thirteen relationship types in the PFTKG, including their labels, quantities, and brief descriptions.

| Type | Labels | Quantity | Description |
| --- | --- | --- | --- |
| Entity | Patient | 473 | Individual receiving perioperative care. |
|  | Laboratory_test | 305 | Clinical laboratory test used for patient assessment. |
|  | Vital_sign | 2711 | Measurement indicating patient's physiological status. |
|  | Disease | 179 | Primary disease diagnosed in the patient. |
|  | Comorbidity | 293 | Coexisting medical condition. |
|  | Surgery | 197 | Surgical procedure performed on the patient. |
|  | Fluid_therapy | 1917 | Fluid therapy protocol. |
|  | Outcome | 415 | Clinical result or endpoint. |
| Relationship | HAS_DISEASE | 380 | Connects Patient entity to Disease entity; indicates the patient has been diagnosed with the disease. |
|  | HAS_COMORBIDITY | 1051 | Connects Patient entity to Comorbidity entity; indicates the patient has a coexisting medical condition. |
|  | RECORDED_LAB_TEST | 305 | Connects Patient entity to Laboratory_test entity; indicates a laboratory test result for the patient. |
|  | RECORDED_VITAL_SIGN | 2711 | Connects Patient entity to Vital_sign entity; indicates a vital sign measurement recorded for the patient. |
|  | RECEIVED_SURGERY | 646 | Connects Patient entity to Surgery entity; indicates the patient underwent the surgical procedure. |
|  | TREATED_WITH_SURGERY | 217 | Connects Disease entity to Surgery entity; indicates the disease was treated with the surgical procedure. |
|  | SUPPORTS_FLUID_THERAPY | 305 | Connects Laboratory_test entity to Fluid_therapy entity; indicates the test supports fluid therapy management. |
|  | RECEIVED_FLUID_THERAPY | 2122 | Connects Patient entity to Fluid_therapy entity; indicates the patient received the fluid therapy. |
|  | GUIDES_FLUID_THERAPY | 2711 | Connects Vital_sign entity to Fluid_therapy entity; indicates the vital sign guides fluid therapy management. |
|  | USES_FLUID_THERAPY | 1944 | Connects Surgery entity to Fluid_therapy entity; indicates fluid therapy was used during the surgical procedure. |
|  | AFFECTS_OUTCOME | 2080 | Connects Fluid_therapy entity to Outcome entity; indicates fluid therapy has an influence on the outcome. |
|  | LEADS_TO_OUTCOME | 534 | Connects Surgery entity to Outcome entity; indicates the surgical procedure resulted in the outcome. |
|  | HAS_OUTCOME | 681 | Connects Patient entity to Outcome entity; indicates the outcome occurred in the patient. |
