# Supplementary material for: Augmenting large language models with clinical knowledge graph for personalized perioperative fluid therapy question answering
Source: PLOS Digit Health. 2026 Jun 11;5(6):e0001474. doi: 10.1371/journal.pdig.0001474 (PMC13257993; doi:10.1371/journal.pdig.0001474)
Supplement: S2 Table — (DOCX) [file pdig.0001474.s006.docx]

**S2 Table.** **Prompt templates for various strategies, knowledge summarization, context selection, and case-based question generation used in the study.**

| **Query Type** | **Prompt Template** |
| --- | --- |
| Knowledge Summarization Level I | You are a clinical AI assistant helping medical researchers to analyze and summarize structured perioperative data, including fluid therapy protocols, vital sign monitoring, patient characteristics, surgical procedures, diseases, comorbidity and clinical outcomes.  # Goal  Write a comprehensive clinical knowledge report based on the provided entities (fluid therapy, vital signs, patients, surgery, diseases, outcomes) and their relationships.  # Report Structure  - TITLE: A concise and descriptive name that summarizes and clearly reflects the unique aspects of the main clinical context.  - SUMMARY: A brief synopsis describing the study or community, including key fluid therapy strategies, patient demographics, disease types, surgical methods, monitored vital signs etc.  - DETAILED FINDINGS: A list of 5-10 key insights about the community. Each insight should have a short summary followed by multiple paragraphs of explanatory text grounded according to the grounding rules below. Be comprehensive.  Return output as a well-formed JSON-formatted string with the following format:      {{          "title": <report_title>,          "summary": <executive_summary>,          "findings": [              {{                  "summary":<insight_1_summary>,                  "explanation": <insight_1_explanation>              }},              {{                  "summary":<insight_2_summary>,                  "explanation": <insight_2_explanation>              }}          ]      }}  # Grounding Rules  Do not include information where the supporting evidence for it is not provided.  Limit the total report length to {max_report_length} words.  # Input example ……  # Output example  …… |
| Knowledge Summarization Level II and III | You are an expert scientific summarizer and report writer. Your task is to review multiple sub-reports, each summarizing different aspects or groups within a broader research study or clinical program. Your goal is to generate a single, comprehensive, and cohesive report that integrates the key findings, methodologies, and implications from all provided sub-reports.  # Instructions  1. Carefully read and integrate the information from all provided sub-reports.  2. Resolve any contradictions or overlapping findings; present the most accurate and consistent synthesis.  3. Structure your output as a single JSON object with the following format:      {{          "title": <A concise, representative title for the overall report>,          "summary": <An executive summary synthesizing the overall study and its main findings>,          "findings": [              {{                  "summary": <Key insight 1>,                  "explanation": <Detailed explanation integrating evidence from the sub-reports>              }},              ...          ]      }}  4. Use clear, scientific, third-person language. Highlight important similarities and differences among sub-reports.  5. Reference specific details or data points from the sub-reports where relevant.  6. Do not invent information not present in the sub-reports.  7. Limit the final description length to {max_length} words.  # Input example ……  # Output example  …… |
| Context selection | Please compare the following three pieces of information and evaluate how helpful each one is for solving the problem. Rate each piece of information on a scale of 1 to 10 (1 means least helpful, 10 means most helpful).  Please output only the scores in the format: x,y,z (corresponding to the scores for Information 1, 2, and 3, numbers only).  questions: {query}  Information 1: {information1}  Information 2: {information2}  Information 3: {information3}  # Example Output:  3,5,7  # Important  **Do not include any other text. Only output numeric commas** |
| Vanilla QA | You are a medical expert specializing in fluid therapy. Answer the following question related to fluid therapy based on your internal knowledge.  Question: {query} |
| CoT QA | You are a medical expert specializing in fluid therapy. Answer the following question related to fluid therapy. First, provide your step-by-step reasoning process. Then, provide the final answer.  Question: {query}  Let's think step by step.  Reasoning:  [Your step-by-step reasoning here]  Final Answer:  [Your answer here] |
| RoT QA | You are a medical expert specializing in fluid therapy.  Question: {query}  Imagine 3 medical experts specializing in fluid therapy are solving this task. Each expert independently provides their step-by-step reasoning and final answer.  After all experts have finished, they discuss together, review and backtrack their previous reasoning steps, and finally reach a consensus on the final answer.  Please present:  [Expert 1's reasoning and answer],  [Expert 2's reasoning and answer],  [Expert 3's reasoning and answer],  [The discussion and the agreed final answer] |
| DocRAG QA | Please answer the fluid therapy question based on the following reference information.  # Instructions:  1. Do not mention that Based on the reference information.  2. If the reference information fails to answer, please complete it based on your internal knowledge.  # Reference Information: {context}  # Question: {query} |
| GraphRAG QA | Please answer the fluid therapy question based on the following reference information.  # Instructions:  1. Do not mention that Based on the reference information.  2. If the reference information fails to answer, please complete it based on your internal knowledge.  # Reference Information: {context}  # Reference Information Confidence: {confidence} (It indicates the reliability of the reference content)  # Question: {query} |
| Preoperative question generation prompt | You are an expert in perioperative fluid therapy, clinical question writing, and medical education. Based on the case information I provide, generate 1 high-quality, discriminative single-best-answer multiple-choice question to assess a large language model’s ability in “understanding individualized perioperative fluid therapy.”  Important notes:  - The stem and options must be constructed using preoperative information only.  - The correct answer and rationale may be determined with reference to the full clinical course, including intraoperative events, postoperative course, and outcome, in order to identify which preoperative judgment was more appropriate for this patient.  - Therefore, this is a “preoperative-scenario stem with an answer supported by the full clinical course” individualized judgment question, rather than a strictly real-time preoperative decision-making question.  Task objective:  Generate 1 high-quality single-best-answer multiple-choice question that mainly evaluates whether the model can make a judgment, based only on preoperative information, that aligns more closely with the real individualized logic of fluid management for this patient. Prioritize assessment of the following abilities:  1. Identifying important preoperative risk factors that influence fluid management  2. Understanding the most important risk balance in this patient’s perioperative fluid management  3. Determining the most critical preoperative monitoring focus or information need  4. Selecting, among several superficially reasonable options, the preoperative judgment best supported by the patient’s full clinical course  Strict requirements:  1. The stem must use preoperative information only. It must not include any intraoperative or postoperative information, imaging conclusions, complications, organ function deterioration, treatment results, or outcome information.  2. The options must also be constructed only from preoperative information and must not directly or indirectly reveal key postoperative clues.  3. The correct answer and rationale may refer to the full clinical course, but the rationale must not directly disclose the full postoperative course; it should only briefly explain why that option better fits the individualized fluid management logic supported by the patient’s subsequent course.  4. Do not generate a question that requires answering based on specific fluid volumes, infusion rates, or explicit execution details of medical orders.  5. The question must have only one best answer.  6. All options must be related to preoperative fluid management; there must be no obviously irrelevant distractors.  7. Incorrect options must have real clinical plausibility: they may be partially supported by some preoperative information, but they fall short because they overlook a more important risk structure, monitoring priority, or individualized balance.  8. Avoid options that are obviously absurd, clearly extreme, absolute, dogmatic, or dismissible without reading the case.  9. The correct answer must not appear obviously correct simply because it sounds more conservative, more vague, more comprehensive, or more like a stock phrase.  10. The question should ideally require integration of at least two preoperative data points for a stable answer, rather than being answerable from a single comorbidity or laboratory value.  11. If the preoperative information itself is insufficient to support a discriminative individualized question, output a blank question.  12. `correct_answer` must contain only A, B, C, or D.  13. `rationale` must be concise and should primarily explain why that option best fits the patient’s individualized fluid management logic; it must not directly disclose specific postoperative events, specific changes in measured values, or the final outcome.  14. The four options should be kept as similar as possible in clinical sophistication, language complexity, and completeness of expression, so that the correct answer does not stand out as an expert summary or guideline-style statement.  15. At least two incorrect options must be “high-level but globally suboptimal” competing judgments: under the preoperative information, they should appear strongly reasonable on the surface, while missing a more important individualized risk or priority.  16. Avoid designing incorrect options as low-quality statements such as “routine management,” “empirically aggressive fluid replacement,” or “fluid strategy has little impact,” unless the other options are written at a similarly plausible clinical level.  17. The correct answer must not appear correct merely through a templated high-level phrasing such as “the key is to balance X and Y with dynamic assessment.” If the correct option uses an advanced or integrative formulation, the other options must be written at a similar level and with similar rhetorical strength.  18. Do not let one option stand out by being obviously longer, more abstract, or more comprehensive; keep option length and complexity as similar as possible.  Preferred question directions for item writing:  1. When multiple preoperative risk factors coexist, which one is most likely to drive this patient’s perioperative fluid management strategy, beyond serving as general background information  2. Which preliminary fluid-management judgment based on preoperative information is most likely to seem reasonable on the surface, yet does not represent the best individualized understanding for this patient  3. If fluid-management judgment is based on only a single preoperative clue, what is the key individualized risk most likely to be overlooked  4. In this patient’s preoperative data, which combination of information should jointly shape perioperative fluid management and should be interpreted together  5. Which preoperative clue is most likely to change the usual perioperative fluid-management approach and create a distinct priority for this patient  6. To avoid a directional error in perioperative fluid-management planning, what information is most worth confirming or prioritizing preoperatively  7. For this patient, which preoperative judgment is most misleading because it is partially reasonable yet globally suboptimal  8. Which preoperative fluid-management focus best matches the individualized logic supported by the patient’s later course, while still requiring more than general common sense  Self-check before output:  - Does the stem contain any intraoperative, postoperative, or outcome information? If so, rewrite it.  - Does any incorrect option appear obviously irrelevant, absurd, or dismissible without reading the case? If so, rewrite it.  - Is the correct answer simply the option that sounds most cautious, most comprehensive, or most like standard phrasing? If so, rewrite it.  - Can the answer be guessed easily from general medical knowledge rather than case-specific details? If so, rewrite it.  - Does the best answer require integration of at least two preoperative data points? If not, rewrite it.  - Does the rationale disclose too much key postoperative information? If so, rewrite it with more restraint.  - Is there a more natural, better balanced, and more competitive answer that is absent from the current four options? If so, rewrite the options.  - Are the four options at a similar level of clinical judgment? If not, rewrite them.  - Does the correct answer become easy to identify because it is clearly longer, more complete, or more like an expert summary? If so, rewrite it.  Please output only in the following JSON format, with no additional text:  {{  "question": "",  "options": {{  "A": "",  "B": "",  "C": "",  "D": ""  }},  "correct_answer": "",  "rationale": ""  }}  [Preoperative information]:  {preoperative_data}  [Full case course information] (for determining the correct answer only; do not disclose directly in the stem or options):  {full_case_data} |
| Intraoperative question generation prompt | You are an expert in perioperative fluid therapy, clinical question writing, and medical education. Based on the case information I provide, generate 1 high-quality, discriminative single-best-answer multiple-choice question to assess a large language model’s ability in “understanding individualized perioperative fluid therapy.”  Important notes:  - The stem and options must be constructed using preoperative information and same-day surgical information only (intraoperative/perioperative day-of-surgery information).  - The correct answer and rationale may be determined with reference to the full clinical course, including the postoperative course and outcome, in order to identify which intraoperative judgment was more appropriate for this patient.  - Therefore, this is a “preoperative + day-of-surgery scenario stem with an answer supported by the full clinical course” individualized judgment question, rather than a strictly real-time closed-loop intraoperative decision-making question.  Task objective:  Generate 1 high-quality single-best-answer multiple-choice question that mainly evaluates whether the model can make a judgment, based only on preoperative information and day-of-surgery information, that aligns more closely with the real individualized logic of fluid management for this patient. Prioritize assessment of the following abilities:  1. Identifying the dominant contradiction in current intraoperative fluid management  2. Determining the intraoperative direction of misjudgment most worth guarding against  3. Understanding the balance among intraoperative fluid administration, hemodynamics, bleeding, comorbidities, and organ perfusion  4. Selecting, among several superficially reasonable options, the intraoperative judgment best supported by the patient’s full clinical course  Strict requirements:  1. The stem must use only preoperative information and day-of-surgery information. It must not include any postoperative information, imaging, complications, organ function deterioration, treatment results, or outcome information.  2. The options must also be constructed only from preoperative information and day-of-surgery information and must not directly or indirectly reveal key postoperative clues.  3. The correct answer and rationale may refer to the full clinical course, but the rationale must not directly disclose the full postoperative course; it should only briefly explain why that option better fits the individualized fluid management logic supported by the patient’s subsequent course.  4. Do not generate a question that requires answering based on specific fluid volumes, infusion rates, or explicit execution details of medical orders.  5. The question must have only one best answer.  6. All options must be related to current intraoperative fluid management; there must be no obviously irrelevant distractors.  7. Incorrect options must have real clinical plausibility: they may be partially supported by some preoperative or intraoperative information, but they fall short because they overlook a more important risk structure, the current dominant contradiction, monitoring priority, or individualized balance.  8. Avoid options that are obviously absurd, clearly extreme, absolute, dogmatic, or dismissible without reading the case.  9. The correct answer must not appear obviously correct simply because it sounds more conservative, more vague, more comprehensive, or more like a stock phrase.  10. The question should ideally require integration of at least two preoperative or day-of-surgery data points for a stable answer, rather than being answerable from a single value, single laboratory result, or single comorbidity.  11. Do not let “further assessment/dynamic assessment” automatically become the correct answer because it sounds safer; it may be the best answer only when the other high-level options are globally weaker.  12. If the preoperative plus day-of-surgery information itself is insufficient to support a discriminative individualized question, output a blank question.  13. `correct_answer` must contain only A, B, C, or D.  14. `rationale` must be concise and should primarily explain why that option best fits the patient’s intraoperative individualized fluid-management logic; it must not directly disclose specific postoperative events, specific changes in measured values, or the final outcome.  15. The four options should be kept as similar as possible in clinical sophistication, language complexity, and completeness of expression, so that the correct answer does not stand out as an expert summary or guideline-style statement.  16. At least two incorrect options must be “high-level but globally suboptimal” competing judgments: under the currently visible information, they should appear strongly reasonable on the surface, while missing a more important individualized risk or priority.  17. Avoid designing incorrect options as low-quality statements such as “routine management,” “continue aggressive fluid replacement,” “fluid strategy has little impact,” or “standard monitoring is sufficient,” unless the other options are written at a similarly plausible clinical level.  18. The correct answer must not appear correct merely through a templated high-level phrasing such as “the key is to balance X and Y with dynamic assessment.” If the correct option uses an advanced or integrative formulation, the other options must be written at a similar level and with similar rhetorical strength.  19. Do not let one option stand out by being obviously longer, more abstract, or more comprehensive; keep option length and complexity as similar as possible.  Preferred question directions for item writing:  1. Under the combined effect of the preoperative background and the current day-of-surgery information, what contradiction is most likely to be driving fluid management at this moment  2. Which fluid-management judgment based on the current day-of-surgery information is most likely to seem reasonable on the surface, yet does not represent the best individualized understanding for this patient  3. If judgment is based only on a single intraoperative clue, such as blood pressure, blood loss, fluid already given, or a laboratory value, what key risk is most likely to be overlooked  4. In this patient’s currently visible information, which combination of preoperative and intraoperative data should jointly shape fluid management and should be interpreted together  5. Which day-of-surgery clue should most strongly redirect an otherwise plausible empiric fluid strategy  6. To avoid a directional error in current fluid management, what judgment focus deserves the highest priority  7. For this patient, which current intraoperative judgment is most misleading because it is partially reasonable yet globally suboptimal  8. Which current fluid-management focus best matches the individualized logic supported by the patient’s later course, while still requiring more than general common sense  Prefer questions that require comparing several superficially reasonable intraoperative judgments, rather than questions that only identify a single risk factor or a single missing piece of information.  Try to avoid designing the item as a direct single-point recognition question such as “which is most important” or “which is most critical,” unless the other options are equally relevant to fluid management and genuinely competitive.  Self-check before output:  - Does the stem contain any postoperative information, imaging, complications, or outcome information? If so, rewrite it.  - Does any incorrect option appear obviously irrelevant, absurd, or dismissible without reading the case? If so, rewrite it.  - Is the correct answer simply the option that sounds most cautious, most comprehensive, or most like standard phrasing? If so, rewrite it.  - Can the answer be guessed easily from general medical knowledge rather than case-specific details? If so, rewrite it.  - Does the best answer require integration of at least two preoperative or intraoperative data points? If not, rewrite it.  - Does the rationale disclose too much key postoperative information? If so, rewrite it with more restraint.  - Is there a more natural, better balanced, and more competitive answer that is absent from the current four options? If so, rewrite the options.  - Are the four options at a similar level of clinical judgment? If not, rewrite them.  - Does the correct answer become easy to identify because it is clearly longer, more complete, or more like an expert summary? If so, rewrite it.  Please output only in the following JSON format, with no additional text:  {{  "question": "",  "options": {{  "A": "",  "B": "",  "C": "",  "D": ""  }},  "correct_answer": "",  "rationale": ""  }}  [Visible preoperative + day-of-surgery information]:  {visible_case_data}  [Full case course information] (for determining the correct answer only; do not disclose directly in the stem or options):  {full_case_data} |
| Postoperative question generation prompt | You are an expert in perioperative fluid therapy, clinical question writing, and medical education. Based on the case information I provide, generate 1 high-quality, discriminative single-best-answer multiple-choice question to assess a large language model’s ability in “understanding individualized perioperative fluid therapy.”  Important notes:  - The stem and options must be constructed using preoperative information, day-of-surgery information, and information available up to the current postoperative day only.  - The correct answer and rationale may be determined with reference to the full clinical course, including later postoperative events and outcome, in order to identify which current judgment was more appropriate for this patient.  - Therefore, this is a “scenario based on information available up to the current postoperative day, with an answer supported by the full clinical course” individualized judgment question, rather than a strictly real-time closed-loop decision question.  Task objective:  Generate 1 high-quality single-best-answer multiple-choice question that mainly evaluates whether the model can make a judgment, based only on preoperative information, day-of-surgery information, and information available up to the current postoperative day, that aligns more closely with the real individualized logic of fluid management for this patient. Prioritize assessment of the following abilities:  1. Identifying the dominant contradiction in current postoperative fluid management  2. Determining the postoperative direction of misjudgment most worth guarding against  3. Understanding the balance among fluid input, current vital signs, laboratory changes, organ perfusion, and the risk of fluid overload  4. Selecting, among several superficially reasonable options, the current judgment best supported by the patient’s full clinical course  Strict requirements:  1. The stem must use only preoperative information, day-of-surgery information, and information available up to the current postoperative day. It must not include any later postoperative-day information, imaging, complications, organ function deterioration outcomes, treatment results, or discharge outcome information.  2. The options must also be constructed only from currently visible information and must not directly or indirectly reveal key later clues.  3. The correct answer and rationale may refer to the full clinical course, but the rationale must not directly disclose the full later postoperative course; it should only briefly explain why that option better fits the individualized fluid management logic supported by the patient’s subsequent course.  4. Do not generate a question that requires answering based on specific fluid volumes, infusion rates, or explicit execution details of medical orders.  5. The question must have only one best answer.  6. All options must be related to current postoperative fluid management; there must be no obviously irrelevant distractors.  7. Incorrect options must have real clinical plausibility: they may be partially supported by currently visible information, but they fall short because they overlook a more important current risk structure, monitoring priority, or individualized balance.  8. Avoid options that are obviously absurd, clearly extreme, absolute, dogmatic, or dismissible without reading the case.  9. The correct answer must not appear obviously correct simply because it sounds more conservative, more vague, more comprehensive, or more like a stock phrase.  10. The question should ideally require integration of at least two currently visible data points for a stable answer, rather than being answerable from a single value, single laboratory result, or single comorbidity.  11. Do not let “further assessment/dynamic assessment” automatically become the correct answer because it sounds safer; it may be the best answer only when the other high-level options are globally weaker.  12. If the currently visible information itself is insufficient to support a discriminative individualized question, output a blank question.  13. `correct_answer` must contain only A, B, C, or D.  14. `rationale` must be concise and should primarily explain why that option best fits the patient’s current postoperative individualized fluid-management logic; it must not directly disclose specific later events, specific changes in measured values, or the final outcome.  15. The four options should be kept as similar as possible in clinical sophistication, language complexity, and completeness of expression, so that the correct answer does not stand out as an expert summary or guideline-style statement.  16. At least two incorrect options must be “high-level but globally suboptimal” competing judgments: under the currently visible information, they should appear strongly reasonable on the surface, while missing a more important current individualized risk or priority.  17. Avoid designing incorrect options as low-quality statements such as “continue routine management,” “continue aggressive fluid replacement,” “fluid strategy has little impact,” or “standard monitoring is sufficient,” unless the other options are written at a similarly plausible clinical level.  18. The correct answer must not appear correct merely through a templated high-level phrasing such as “the key is to balance X and Y with dynamic assessment.” If the correct option uses an advanced or integrative formulation, the other options must be written at a similar level and with similar rhetorical strength.  19. Do not let one option stand out by being obviously longer, more abstract, or more comprehensive; keep option length and complexity as similar as possible.  Preferred question directions for item writing:  1. Under the combined effect of the preoperative background, day-of-surgery information, and current postoperative information, what contradiction is most likely to be driving fluid management at this moment  2. Which fluid-management judgment based on the current postoperative information is most likely to seem reasonable on the surface, yet does not represent the best individualized understanding for this patient  3. If judgment is based only on a single current clue, such as blood pressure, creatinine, cumulative fluid given, albumin, urine output, or a laboratory value, what key risk is most likely to be overlooked  4. In this patient’s currently visible information, which group of findings should most strongly shape fluid management together and should be interpreted jointly  5. Which current clue should most strongly redirect the existing fluid-management strategy  6. To avoid a directional error in current fluid management, what judgment focus deserves the highest priority  7. For this patient, which current postoperative judgment is most misleading because it is partially reasonable yet globally suboptimal  8. Which current fluid-management focus best matches the individualized logic supported by the patient’s later course, while still requiring more than general common sense  Prefer questions that require comparing several superficially reasonable current judgments, rather than questions that only identify a single risk factor or a single missing piece of information.  Try to avoid designing the item as a direct single-point recognition question such as “which is most important” or “which is most critical,” unless the other options are equally relevant to fluid management and genuinely competitive.  Self-check before output:  - Does the stem contain any later postoperative-day information, imaging, complications, or outcome information? If so, rewrite it.  - Does any incorrect option appear obviously irrelevant, absurd, or dismissible without reading the case? If so, rewrite it.  - Is the correct answer simply the option that sounds most cautious, most comprehensive, or most like standard phrasing? If so, rewrite it.  - Can the answer be guessed easily from general medical knowledge rather than case-specific details? If so, rewrite it.  - Does the best answer require integration of at least two currently visible data points? If not, rewrite it.  - Does the rationale disclose too much key later information? If so, rewrite it with more restraint.  - Is there a more natural, better balanced, and more competitive answer that is absent from the current four options? If so, rewrite the options.  - Are the four options at a similar level of clinical judgment? If not, rewrite them.  - Does the correct answer become easy to identify because it is clearly longer, more complete, or more like an expert summary? If so, rewrite it.  Please output only in the following JSON format, with no additional text:  {{  "question": "",  "options": {{  "A": "",  "B": "",  "C": "",  "D": ""  }},  "correct_answer": "",  "rationale": ""  }}  [Information available up to the current postoperative day]:  {visible_case_data}  [Full case course information] (for determining the correct answer only; do not disclose directly in the stem or options):  {full_case_data} |
