# Supplementary material for: Augmenting large language models with clinical knowledge graph for personalized perioperative fluid therapy question answering
Source: PLOS Digit Health. 2026 Jun 11;5(6):e0001474. doi: 10.1371/journal.pdig.0001474 (PMC13257993; doi:10.1371/journal.pdig.0001474)
Supplement: S3 Table — Overview of the five core clinical categories used for the design and classification of the personalized fluid therapy question dataset, including representative content for each category. (DOCX) [file pdig.0001474.s007.docx]

**S3 Table. Question Dataset Categories**. Overview of the five core clinical categories used for the design and classification of the personalized fluid therapy question dataset, including representative content for each category.

| **Category** | **Description** |
| --- | --- |
| General Fluid Therapy Principles | Common knowledge related to the basic theories, indications, contraindications, and selection criteria of fluid therapy. |
| Patient-specific Fluid Management | Individualized fluid management strategies for different underlying diseases, surgical types, and special patient populations. |
| Hemodynamic Monitoring and Management | Focuses on hemodynamic monitoring, interpretation of key parameters, and corresponding interventions during fluid therapy. |
| Electrolyte Solution Management | Focused on the identification and management of electrolyte disturbances and the impact of fluid selection on electrolyte balance. |
| Vasoactive and Transfusion Management | Guidance for decision-making and key considerations regarding vasoactive drugs and transfusion during fluid management. |
