# Supplementary material for: Augmenting large language models with clinical knowledge graph for personalized perioperative fluid therapy question answering
Source: PLOS Digit Health. 2026 Jun 11;5(6):e0001474. doi: 10.1371/journal.pdig.0001474 (PMC13257993; doi:10.1371/journal.pdig.0001474)
Supplement: S4 Table — As the original source data were in Chinese, the case-based questions were likewise formulated in Chinese. (DOCX) [file pdig.0001474.s008.docx]

**S4 Table. Representative examples from the question dataset.** As the original source data were in Chinese, the case-based questions were likewise formulated in Chinese.

| **Question set** | **Multiple-choice question** | **Open-ended question** | **Reference answer** |
| --- | --- | --- | --- |
| Knowledge-based question set | In pediatric surgery, when the cumulative dose of isotonic crystalloid (e.g., acetate Ringer’s or lactated Ringer’s) reaches or exceeds 25 ml/kg, which replacement fluid should be used next?  A. Continue unrestricted isotonic crystalloid;  B. Begin a 1:1 switch to 5% albumin;  C. Switch to hypotonic saline;  D. Switch to 5% albumin in an isotonic colloid-balanced ratio. | In pediatric surgery, when the cumulative dose of isotonic crystalloid (e.g., acetate Ringer’s or lactated Ringer’s) reaches or exceeds 25 ml/kg, which replacement fluid should be used next? | Switch to 5% albumin in an isotonic colloid-balanced ratio. |
| Case-based question set | 病例背景：**岁**；计划性手术；术前诊断：**；合并**；术前生命体征：BP **/** mmHg，MAP约** mmHg，HR约**次/分，SpO2约**；实验室：Hb 121.66666666666669 g/L，Plt 99.66666666666669×10^9/L，Cr 78.8 μmol/L，Alb 37.72 g/L，TBil 20.3 μmol/L，ALT 190.8 U/L，Na+ 139.3 mmol/L，K+ 3.86 mmol/L  问题：**岁**性，拟行**，术前诊断为**，合并**。术前生命体征平稳：MAP约** mmHg，心率约**次/分，SpO2正常。实验室检查示：Hb约122 g/L，血小板约100×10^9/L，Cr约79 μmol/L，白蛋白约37.7 g/L，总胆红素约20 μmol/L，ALT约191 U/L，电解质基本正常。基于这些术前信息，以下哪一项最符合该患者围术期液体治疗中应优先把握的个体化判断？  A. 术前循环、肾功能及电解质总体稳定，当前液体策略宜首先围绕维持有效循环容量和重要器官灌注进行个体化评估，在此基础上再根据肝功能异常程度适度调整，而不必预设明显限制性的容量取向  B. 虽无明显低白蛋白或休克表现，但因拟行**且已有肝细胞损伤和血小板偏低，术前更应关注避免容量过多导致残余肝脏及手术相关不利影响，而不是把其视为常规需积极预充的稳定患者  C. **且血小板处于偏低边缘，提示围术期止血与容量管理需协同考虑，因此术前液体治疗更应强调维持相对稳定的血液浓缩状态，避免过早稀释性影响，而灌注目标可适当从属于这一原则  D. 考虑高龄合并**，术前液体评估应更重视其可能较窄的灌注安全窗，因此宜采取偏积极但不过量的容量准备，以尽量减少麻醉诱导后血压下降对脑、心、肾等重要脏器灌注的不利影响 | | B. 虽无明显低白蛋白或休克表现，但因拟行**且已有肝细胞损伤和血小板偏低，术前更应关注避免容量过多导致残余肝脏及手术相关不利影响，而不是把其视为常规需积极预充的稳定患者 |
