# Supplementary material for: Augmenting large language models with clinical knowledge graph for personalized perioperative fluid therapy question answering
Source: PLOS Digit Health. 2026 Jun 11;5(6):e0001474. doi: 10.1371/journal.pdig.0001474 (PMC13257993; doi:10.1371/journal.pdig.0001474)
Supplement: S5 Table — (DOCX) [file pdig.0001474.s009.docx]

**S5 Table. McNemar test results for GraphRAG versus the nurse baseline on the knowledge-based question set.**

| **Question Set** | **LLM** | **p-value** | **Significance** |
| --- | --- | --- | --- |
| Multiple-choice | Claude | 0.0324 | Significant |
| Multiple-choice | Gemini | 0.0752 | Not significant |
| Multiple-choice | GPT-4o | 0.1508 | Not significant |
| Open-ended | Claude | 1.55e-08 | Significant |
| Open-ended | Gemini | 1.59e-11 | Significant |
| Open-ended | GPT-4o | 6.72e-13 | Significant |
