# Supplementary material for: Augmenting large language models with clinical knowledge graph for personalized perioperative fluid therapy question answering
Source: PLOS Digit Health. 2026 Jun 11;5(6):e0001474. doi: 10.1371/journal.pdig.0001474 (PMC13257993; doi:10.1371/journal.pdig.0001474)
Supplement: S6 Table — (DOCX) [file pdig.0001474.s010.docx]

To further assess deployment feasibility, we compared the offline preprocessing cost and storage requirements of DocRAG and GraphRAG. Preprocessing time refers to the time required to build the retrieval index before inference. For DocRAG, this included document preprocessing, embedding generation, and document-level vector index construction. For GraphRAG, this included hierarchical community detection, recursive community summarization, embedding generation, and construction of the hierarchical finding-level summary vector index. Storage requirement refers to the total on-disk size of the retrieval index used at inference time. All measurements were obtained in the same computing environment: 16 vCPUs of Intel(R) Xeon(R) Platinum 8352V CPU, 128 GB RAM, and Ubuntu 22.04, using CPU only. Because a substantial proportion of the preprocessing time arose from LLM API calls during summarization, the reported values should be interpreted as practical end-to-end index-construction cost estimates rather than purely local computational overhead.

**S6 Table. Preprocessing cost and storage requirements of DocRAG and GraphRAG.**

| **Method** | **Preprocessing time (min)** | **Storage requirement (MB)** | **Index type** |
| --- | --- | --- | --- |
| DocRAG | 3.38 | 146.08 | Document-level vector index |
| GraphRAG | 618.92 | 274.23 | Hierarchical finding-level summary vector index |
