# Supplementary material for: Augmenting large language models with clinical knowledge graph for personalized perioperative fluid therapy question answering
Source: PLOS Digit Health. 2026 Jun 11;5(6):e0001474. doi: 10.1371/journal.pdig.0001474 (PMC13257993; doi:10.1371/journal.pdig.0001474)
Supplement: S7 Table — (DOCX) [file pdig.0001474.s011.docx]

**S7 Table**. **Comparison of the present framework with representative GraphRAG-related and knowledge-graph–augmented retrieval approaches.** The table summarizes the knowledge source, retrieval unit, and core retrieval mechanism of the present framework and several representative prior approaches, including Microsoft GraphRAG, G-Retriever, LightRAG, Cypher-based RAG, and vector-based KG retrieval. This comparison clarifies which components of the present study build on prior GraphRAG-related ideas and which aspects were specifically adapted for personalized perioperative fluid therapy question answering.

| Methods | Knowledge Source | Retrieval Unit | Core mechanism |
| --- | --- | --- | --- |
| GraphRAG (ours) | Pre-built domain knowledge graph (PFTKG) | Finding-level chunks from community summaries | Performs hierarchical community detection over a clinical KG, recursively generates summaries across three community levels, and vectorizes fine-grained findings from the summaries as a hierarchical index. At query time, it combines similarity-based retrieval with LLM reranking to select the most relevant context for answer generation. |
| DocRAG | Unstructured document corpus | Document chunks | Splits source documents into chunks and builds a vector index; at query time, retrieves relevant text chunks based on semantic similarity. |
| GraphRAG([1](#_ENREF_1)) | Unstructured document corpus | Community summaries | Uses an LLM to extract entities and relations from documents and construct a graph index, then performs hierarchical community detection and recursive community summarization. During querying, it generates globally oriented answers by synthesizing information from multiple community summaries. |
| G-Retriever([2](#_ENREF_2)) | Text-attributed graph | Subgraphs | Semantically encodes the textual attributes of graph nodes and edges. At query time, it retrieves relevant graph elements based on similarity between the query and node/edge text attributes, organizes them into coherent subgraphs using graph structure, and feeds both the textualized subgraphs and graph representations into an LLM to generate answers. |
| LightRAG([3](#_ENREF_3)) | Unstructured document corpus | Low-level entities/relations and high-level topic information | Uses an LLM to extract entities and relations from documents and build a lightweight graph index. At query time, it first extracts low-level and high-level keywords, then matches relevant entities, relations, and topic information via vector similarity, and combines graph neighborhood expansion for answer generation. |
| Cypher RAG([4](#_ENREF_4)) | Pre-built domain knowledge graph | Structured graph query results (nodes, relations, paths) | Converts the user query into a graph database query statement, precisely retrieves relevant entities, relations, and paths from the knowledge graph, and provides the retrieved results as structured context to the LLM for answer generation. |
| Vector RAG([4](#_ENREF_4)) | Pre-built domain knowledge graph | Similar nodes and their neighborhood subgraphs | First identifies domain entities in the query, then retrieves the most semantically relevant nodes and their neighborhood structures based on vector representations of graph entities and relations, and uses the relevant subgraphs as context for LLM-based answer generation. |

1. Edge D, Trinh H, Cheng N, Bradley J, Chao A, Mody A, et al. From local to global: A graph rag approach to query-focused summarization. arXiv preprint arXiv:240416130. 2024.

2. He X, Tian Y, Sun Y, Chawla NV, Laurent T, LeCun Y, et al. G-retriever: Retrieval-augmented generation for textual graph understanding and question answering. Advances in Neural Information Processing Systems. 2024;37:132876-907.

3. Guo Z, Xia L, Yu Y, Ao T, Huang C. Lightrag: Simple and fast retrieval-augmented generation. arXiv preprint arXiv:241005779. 2024;2(3).

4. Song J, Xu Z, He M, Feng J, Shen B. Graph retrieval augmented large language models for facial phenotype associated rare genetic disease. NPJ Digital Medicine. 2025;8(1):543.
