# Supplementary material for: Augmenting large language models with clinical knowledge graph for personalized perioperative fluid therapy question answering
Source: PLOS Digit Health. 2026 Jun 11;5(6):e0001474. doi: 10.1371/journal.pdig.0001474 (PMC13257993; doi:10.1371/journal.pdig.0001474)
Supplement: S1 Appendix — Definitions and formulas for modularity and communitude used to compare community detection algorithms. (DOCX) [file pdig.0001474.s012.docx]

To evaluate the quality of partitions produced by different community detection algorithms, this paper adopts two metrics: Modularity [[5](#_ENREF_5)] and Communitude [[6](#_ENREF_6)]. Modularity measures whether the overall community partition exhibits the structural property of dense intra-community connections and sparse inter-community connections. Communitude measures the statistical significance of an individual community relative to a random background, namely whether its internal connectivity is significantly stronger than the random expectation. Since this paper focuses on the overall partitioning results produced by the algorithms, it further computes the average Communitude over all detected communities. Higher values of these two metrics indicate better community partitioning results.

Let $G=(V,E)$, where $V$ is the set of nodes, $E$ is the set of edges, $n=\left| V \right|$ is the number of nodes, and $m=\left| E \right|$ is the number of edges. For any node set $S\subseteq V$, let $e\left[ S \right]$ denote the number of internal edges in $S$*,* and let $D\left[ S \right]$ denote the sum of the degrees of all nodes in $S$ in the original graph. Let $\mathbb{C=\{}C_{1}, C_{2},\ldots,C_{k}\}$ denote the set of communities in a partition of the graph.

Modularity is defined as:

$$Q\left( \mathbb{C} \right)=\sum_{C_{i}\in\mathbb{C}} \left( \frac{e\left[ C_{i} \right]}{m}-\left( \frac{D\left[ C_{i} \right]}{2m} \right)^{2} \right)$$

where $e\left[ C_{i} \right]$ denotes the number of internal edges in community $C_{i}$, and $D\left[ C_{i} \right]$  denotes the sum of the degrees of the nodes in $C_{i}$.

For any node set $S\subseteq V$, its Communitude is defined as:

$$communitude(S)=\frac{\frac{e\left[ S \right]}{m}-\left( \frac{D\left[ S \right]}{2m} \right)^{2}}{\sqrt{\left( \frac{D\left[ S \right]}{2m} \right)^{2}\left( 1-\left( \frac{D\left[ S \right]}{2m} \right)^{2} \right)}}$$

For $S=\emptyset$ or $S=V$ , define $\text{communitude}(S)=0$. This paper further defines the Communitude of the overall partition as the average Communitude of all communities:

$$AvgCommunitude\left( \mathbb{C} \right)=\frac{1}{k}\sum_{i=1}^{k} communitude(C_{i})$$

where $k$ is the total number of communities, and $C_{i}$ is the $i$-th community.
