# Supplementary material for: Augmenting large language models with clinical knowledge graph for personalized perioperative fluid therapy question answering
Source: PLOS Digit Health. 2026 Jun 11;5(6):e0001474. doi: 10.1371/journal.pdig.0001474 (PMC13257993; doi:10.1371/journal.pdig.0001474)
Supplement: S2 Appendix — Detailed implementation of GraphRAG preprocessing and inference, including pseudocode. (DOCX) [file pdig.0001474.s013.docx]

We applied the EdMot algorithm to the PFTKG for community detection, automatically dividing the graph into several topic-specific knowledge communities based on semantic and clinical relevance. Each community focuses on specific clinical topic or treatment scenarios, and this grouping approach significantly improves the efficiency of knowledge organization by enabling effective aggregation of similar information.

The entire KG was divided into three hierarchical layers of communities. The first-layer communities are at the top level and contain several second-layer communities; each second-layer community is further subdivided into multiple third-layer communities. At each level, communities are automatically aggregated based on semantic and clinical relevance, forming tightly structured and recursively organized knowledge units. The third-layer communities directly contain specific entities and relationships from the KG. **S3 Fig** illustrates the distribution of the detected first-layer communities, where clinically related entities are automatically grouped into the same community.

Subsequently, we used an LLM to perform recursive knowledge summarization on these communities. The core purpose of this approach is to fully leverage the hierarchical and progressive structure of the communities, enabling more systematic knowledge summarization and supporting multi-level knowledge acquisition needs. The specific process is as follows:

- Third-layer community summarization: We input the entities and relationships (triples) contained in each third-layer community into the LLM. The LLM then summarizes the fine-grained knowledge within the community, extracting the specific clinical knowledge or key treatment points that the community focuses on.
- Second-layer community summarization: Building upon the knowledge summaries of the third-layer communities, the LLM further summarizes each second-layer community, integrating the multiple fine-grained knowledge points it covers to form higher-level clinical topics or scenario descriptions.
- First-layer community summarization: Finally, based on the knowledge summaries of all second-layer communities, the LLM summarizes and generalizes the content at the first-layer community level, forming a macro-level clinical knowledge pathway.

Through this recursive knowledge summarization process, the LLM is able to progressively integrate and refine knowledge at each level, achieving multi-level knowledge fusion from fine to coarse, and from concrete to abstract. After performing recursive knowledge summarization on multi-level communities, we employed OpenAI’s text-embedding-3-small model to vectorize the summarizations at each level, constructing a hierarchical vector database for semantic similarity-based retrieval in GraphRAG. The overall GraphRAG preprocessing procedure is summarized in **Algorithm 1**.

When a user submits a clinical question, the system vectorizes the query, retrieves the top three relevant contexts using cosine similarity, and leverages the LLM to select the most suitable context for response generation. In DocRAG, document vectors are used for direct similarity-based retrieval and response generation. For both approaches, the final response is generated by inputting the selected context together with the user query into the LLM to generate a personalized fluid therapy response. The GraphRAG inference procedure is summarized in **Algorithm 2**. Details of the LLM prompt templates used for summarization, context selection, and response generation are provided in S2 Table.

| **Algorithm 1**. GraphRAG preprocessing |
| --- |
| Input:  G = personalized fluid therapy knowledge graph  max_depth = maximum number of community layers  min_size = minimum number of nodes required for further partition  LLM_sum = GPT-4.1-2025-04-14  Emb = text-embedding-3-small  Output:  CTree = hierarchical community tree with community reports  VDB = vector database of multi-level knowledge chunks  Notation:  G_sub = a subgraph of G  nodes(G_sub) = the set of nodes contained in G_sub  path = the hierarchical location of a community in CTree  report = the summary generated for one community  finding_i = the i-th finding item in a community report  1: function RecursiveCommunityDetection(G_sub, depth):  2: Initialize SubTree as an empty community container  3: if depth = max_depth or the number of nodes in G_sub is smaller than min_size then  4: return empty  5: end if  6: Apply EdMot to G_sub to partition it into communities  7: for each detected community c do  8: Extract the nodes belonging to c from G_sub  9: Build the subgraph G_c for community c  10: Store the node set of c in SubTree[c]  11: SubTree[c].sub ← RecursiveCommunityDetection(G_c, depth + 1)  12: end for  13: return SubTree  14: end function  15: CTree ← RecursiveCommunityDetection(G, 0)  16: Enumerate all leaf-community paths in CTree  17: for each path p do  18: Extract all triples contained in the leaf community at p  19: Convert these triples into textual relation statements  20: Use gpt-4.1-2025-04-14 to generate a structured report  21: Store the report at path p in CTree  22: end for  23: Enumerate all second-layer community paths in CTree  24: for each path p do  25: Collect the reports of all direct child communities of p  26: Use gpt-4.1-2025-04-14 to synthesize them into one parent report  27: Store the parent report at path p in CTree  28: end for  29: Enumerate all first-layer community paths in CTree  30: for each path p do  31: Collect the reports of all direct child communities of p  32: Use gpt-4.1-2025-04-14 to synthesize them into one parent report  33: Store the parent report at path p in CTree  34: end for  35: Initialize an empty chunk list X  36: Traverse all communities in CTree  37: for each community path p do  38: Read the report stored at p  39: for each finding_i in the report do  40: Construct chunk_text by concatenating the summary and explanation of finding_i  41: Store chunk_text together with metadata {path, finding index}  42: Append the chunk to X  43: end for  44: end for  45: Encode each chunk_text in X using text-embedding-3-small  46: Insert all chunk embeddings and metadata into Chroma  47: Return CTree and VDB |

| **Algorithm 2**. GraphRAG inference |
| --- |
| Input:  q = user query  CTree = hierarchical community tree  VDB = hierarchical vector database  LLM = target large language model  Emb = text-embedding-3-small  Output:  a = final answer  1: Encode q using text-embedding-3-small.  2: Retrieve the top three relevant finding contexts from VDB according to cosine similarity between the query embedding and the stored community finding embeddings.  3: Construct a context-selection prompt using q and the three retrieved contexts.  4: Use LLM to evaluate the retrieved contexts and select the most suitable context for q.  5: Construct the final question-answering prompt using the selected context and q.  6: Use LLM to generate the final answer a.  7: Return a. |
