# Supplementary material for: Augmenting large language models with clinical knowledge graph for personalized perioperative fluid therapy question answering
Source: PLOS Digit Health. 2026 Jun 11;5(6):e0001474. doi: 10.1371/journal.pdig.0001474 (PMC13257993; doi:10.1371/journal.pdig.0001474)
Supplement: S3 Appendix — Detailed evaluation procedures for accuracy, honesty, error composition, human reference comparison, and response length. (DOCX) [file pdig.0001474.s014.docx]

We evaluated three state-of-the-art LLMs: GPT-4o (gpt-4o-2024-08-06), Claude Opus 4 (Claude-Opus-4-20250514), and Gemini 2.5 Pro (Gemini-2.5-Pro). All LLMs were accessed via API with a temperature of 0.3 for reproducibility. GraphRAG and DocRAG were compared with several prompting strategies:

1. **Vanilla:** The basic LLM response, generated without any specialized prompting or reasoning instructions.
2. **CoT:** Guides the LLM to perform reasoning by encouraging "step-by-step thinking".
3. **RoT:** Breaks the task into multiple steps, simulating discussion and multidisciplinary consultation to reach a final answer.
4. **DocRAG:** Basic RAG technique that uses simple vector retrieval from PFTKB as the only data source, without any additional methods.
5. **GraphRAG:** Organizes PFTKB by community detection and recursive summarization; retrieves and selects relevant hierarchical contexts for response generation.

Full prompt templates are in **S2 Table**.

Overall accuracy: We compared the overall accuracy of the following methods: Vanilla LLM, CoT, RoT, DocRAG, and GraphRAG. These methods were evaluated on question dataset we constructed for personalized fluid therapy. The evaluation process involved two clinicians with expertise in perioperative care independently comparing the output of each method with the reference answers according to predefined scoring criteria. Disagreements were resolved through joint review and consensus adjudication. To provide a human baseline for comparison, we also included answers from a nurse with over 5 years of perioperative care experience (Jinhua Feng) who completed the same question dataset. Importantly, she answered the open-ended questions first, followed by the multiple-choice questions, to avoid any potential influence from the provided options. Her responses were evaluated using the same accuracy criteria as applied to the LLM outputs.

Honesty: To evaluate the honesty of the LLMs, experts annotated its incorrect answers. If the LLM explicitly indicated that it did not know the answer, the response was labeled as honest; conversely, if the LLM fabricated an answer, it was labeled as dishonest. Honesty is particularly important in the medical domain, where it is crucial for LLM to admit uncertainty rather than generate fabricated answers, as incorrect or made-up responses may lead to serious consequences. In addition, we added a "Don’t Know" option to the multiple-choice questions to further encourage the LLM to express uncertainty honestly when appropriate. The inclusion of this option allows for a clearer assessment of the LLM’s response to uncertain questions and encourages the LLM to refrain from answering when unsure, thereby reducing the generation of misinformation.

Error composition: To investigate the error patterns of the LLM, all incorrect responses underwent manual error analysis. Two clinicians with over 5 years of experience in perioperative care classified each error into one of four mutually exclusive categories:

1. **Question misinterpretation:** Whether the LLM fundamentally misunderstood the intent, scope, or requirements of the question.
2. **Insufficient internal knowledge:** If the question was correctly understood, the evaluator then assessed whether the LLM’s response lacked the essential factual knowledge required for a correct answer. If key knowledge was missing, the error was categorized as insufficient internal knowledge. During the RAG process, if the correct context was provided but the LLM failed to utilize the key information in the context and instead relied on its internal knowledge, this was also classified as insufficient internal knowledge.
3. **Reasoning error:** If the response indicated that the LLM possessed the correct knowledge but made a mistake in reasoning, logic, or calculation, leading to an incorrect conclusion, the error was categorized as reasoning error. During the RAG process, if the LLM leveraged the key information from the context but reasoned incorrectly, this error was also classified as reasoning error.
4. **Context irrelevant (specific to RAG):** For answers generated using DocRAG or GraphRAG if the error was caused by the LLM relying on retrieved contexts that were irrelevant to the question, the error was categorized as "Irrelevant Context".

This structured protocol ensures that all observed errors are classified consistently and logically, helping us better understand and improve the capabilities and performance of LLMs.

Response length: To evaluate the computational cost and verbosity of each LLM and prompting strategy, we measured the length of generated responses by calculating the token counts. To ensure consistency and comparability across all experiments, a unified tokenization method was adopted. Specifically, tokenization was performed using the tiktoken library with the cl100k_base encoding. This encoding is widely used by modern LLMs, making it a suitable standard for cross-LLM comparisons. This analysis ensures that response length is measured in a consistent and reliable manner.
